# Metagenomic analysis unveils the microbial landscape of pancreatic tumors

**DOI:** 10.3389/fmicb.2023.1275374

**Published:** 2023-12-21

**Authors:** Sheema Khan, Goutam Banerjee, Saini Setua, Daleniece Higgins Jones, Bhavin V. Chauhan, Anupam Dhasmana, Pratik Banerjee, Murali Mohan Yallapu, Stephen Behrman, Subhash C. Chauhan

**Affiliations:** ^1^Department of Immunology and Microbiology, School of Medicine, University of Texas Rio Grande Valley, Edinburg, TX, United States; ^2^South Texas Center of Excellence in Cancer Research, School of Medicine, the University of Texas Rio Grande Valley, McAllen, TX, United States; ^3^Department of Food Science and Human Nutrition, University of Illinois at Urbana-Champaign, 105 Agricultural Bioprocess Laboratory, Urbana, IL, United States; ^4^Department of Pharmaceutical Sciences, College of Pharmacy, University of Tennessee Health Science Center, Memphis, TN, United States; ^5^Center for Blood Oxygen Transport and Hemostasis (CBOTH), Department of Pediatrics, University of Maryland, Baltimore, MD, United States; ^6^Division of Epidemiology, Biostatistics, and Environmental Health, University of Memphis, Memphis, TN, United States; ^7^Department of Public Health, University of Tennessee, Knoxville, TN, United States; ^8^Department of Surgery, Baptist Memorial Hospital and Medical Education, Memphis, TN, United States

**Keywords:** microbiota, metagenomics, pancreatic ductal adenocarcinoma, PD-L1, Tissues

## Abstract

The composition of resident microbes in the human body is linked to various diseases and their treatment outcomes. Although studies have identified pancreatic ductal adenocarcinoma (PDAC)-associated bacterial communities in the oral and gut samples, herein, we hypothesize that the prevalence of microbiota in pancreatic tumor tissues is different as compared with their matched adjacent, histologically normal appearing tissues, and these microbial molecular signatures can be highly useful for PDAC diagnosis/prognosis. In this study, we performed comparative profiling of bacterial populations in pancreatic tumors and their respective adjacent normal tissues using 16S rRNA-based metagenomics analysis. This study revealed a higher abundance of Proteobacteria and Actinomycetota in tumor tissues compared with adjacent normal tissues. Interestingly, the linear discriminant analysis (LDA) scores unambiguously revealed an enrichment of Delftia in tumor tissues, whereas Sphingomonas, Streptococcus, and Citrobacter exhibited a depletion in tumor tissues. Furthermore, we analyzed the microbial composition between different groups of patients with different tumor differentiation stages. The bacterial genera, Delftia and Staphylococcus, were very high at the G1 stages (well differentiated) compared with G2 (well to moderate/moderately differentiated) and G3/G4 (poorly differentiated) stages. However, the abundance of Actinobacter and Cloacibacterium was found to be very high in G2 and G3, respectively. Additionally, we evaluated the correlation of programmed death-ligand (PDL1) expression with the abundance of bacterial genera in tumor lesions. Our results indicated that three genera such as Streptomyces, Cutibacterium, and Delftia have a positive correlation with PD-L1 expression. Collectively, these findings demonstrate that PDAC lesions harbor relatively different microbiota compared with their normal tumor adjacent tissues, and this information may be helpful for the diagnosis and prognosis of PADC patients.

## 1 Introduction

Pancreatic ductal adenocarcinoma (PDAC) is a highly lethal disease (Riquelme et al., 2019), and a minor subset of patients survive more than 5 years after surgery (Dal Molin et al., 2015); the factors that determine such enigmatic long-term survival are unknown. Management of PDAC is exceptionally difficult due to late diagnosis, anatomical location, vague and non-specific symptoms, lack of sensitive and specific biomarkers, poor response to available therapeutic modalities, and drug resistance. No specific molecular markers and/or imaging technologies have the sensitivity or specificity to identify patients at an early stage of the disease or with a high risk of developing PDAC and regard them as candidates for early surgical intervention. Therefore, there is a need to develop discriminatory, non-invasive molecular markers for identifying early-stage PDAC and its precursor lesions, pancreatic intraepithelial neoplasia-II-III (PanIN-II-III), and mucinous pancreatic cystic neoplasms (IPMNs/MCNs). Recent studies suggest a critical role of microbiome in cancer progression (Yatsunenko et al., 2012; Davenport et al., 2015) and prevention (Bultman, 2016). Typically, the microbiome constitutes approximately 3% of the total body weight (Sender et al., 2016), and alterations in microbial species can be a significant indicator of the diseased stage and disease progression. Human gut microbiota varies extensively between individuals (Turnbaugh et al., 2009; Qin et al., 2010; Huse et al., 2012), which frequently associates with diet (Muegge et al., 2011; Wu et al., 2011; David et al., 2014), age (Yatsunenko et al., 2012; Davenport et al., 2015), sex (Fierer et al., 2008; Davenport et al., 2015), body mass index (BMI) (Turnbaugh et al., 2009; Structure, 2012), and disease presentation (Zackular et al., 2013; Walters et al., 2014) and can influence the balance between beneficial and pathogenic bacterial species (Almeida et al., 2006). The microbiome in bodily fluids and organs is altered with diverse health outcomes, including transformed immunity, obesity, cancer, and diabetes (Ley, 2010; Kau et al., 2011). However, the connection between microbial infection, oral health, and PDAC in the realm of disease dynamics is not well studied.

Recently, the microbiome composition has emerged as a potential driving force for identifying early cancer biomarkers (Kostic et al., 2012; Zambirinis et al., 2014). Altered gut and oral microbiota have been recently studied to determine the association of microbiome with the pathogenesis of PDAC. Studies have reported their involvement in mediating tumor responses to chemotherapy and immunotherapy in patients with melanoma and lung cancers (Gopalakrishnan et al., 2018; Matson et al., 2018; Routy et al., 2018). A recent study found the presence of *Fusobacterium* species in formalin-fixed, paraffin-embedded (FFPE) tissue specimens of PDAC patients (Mitsuhashi et al., 2015). Previous studies have reported the associations between periodontal disease pathogens and pancreatic cancer risk, especially *Porphyromonas gingivalis* (Liu et al., 2019). Periampullary and PDAC cancers were found to have reduced *Lactobacillus* spp. compared with healthy pancreas or bearing pancreatic cysts (Del Castillo et al., 2019). Another very broad study using specimens from the pancreas, bile, and jejunum was conducted in pancreatic cancer patients who underwent pancreaticoduodenectomy and pancreatic cysts. Bacterial taxa that were present in the pancreatic ducts and the bile duct are *Prevotella, Haemophilus, Aggregatibacter*, and *Fusobacterium* (Rogers et al., 2017). *Bifidobacterium* spp. and *L. acidophilus* downregulated the expression of oncomiRs (miR-155 and miR-221) and reduced KRAS mutations in the liver tissue (Pushalkar et al., 2018). These studies provide an understanding of the potential bacterial translocation from the intestinal tract into the peritumoral milieu, which is more relevant to disease development. However, the analysis of microbial communities in gut or oral samples may reflect a disease state, without fully reflecting the tumor microenvironment, tissue-adhering bacteria, and topology of the diseased pancreas. Thus, the relevance of microbiota composition found in the human PDAC can only be studied by comparing it with the surrounding non-malignant tissues of the pancreas from the same patient. Microbiota composition is important to understand what microbial genera/species are required to investigate for their favorable or adverse contribution to the natural history of pancreatic cancer, which remains incompletely studied.

The breakthrough success of anti–PD-1/PD-L1 immune-checkpoint inhibitor (ICI) treatment has been witnessed in various cancers such as advanced-stage melanoma, non-small cell lung cancer (NSCLC), and renal cell cancer (RCC) (Hargadon et al., 2018). Despite this, only a minority of patients (10–40%); (Topalian et al., 2012; Borghaei et al., 2015; Motzer et al., 2015) experience the benefits of ICI therapy. The downregulation of PD-L1 expression is believed to be a key factor contributing to the limited effectiveness of anti-PD-L1 ICIs in PDAC treatment. Emerging evidence underscores the pivotal role of the gut microbiota in supporting ICI treatment in advanced melanoma, NSCLC, RCC, and urothelial cancer (Chaput et al., 2017; Routy et al., 2018). The identification of specific bacterial taxa associated with PD-L1 expression in cancers holds immense potential for developing a microbiome-based combinatory treatment strategy. This approach aims to enhance the overall response rate to anti–PD-1/PD-L1 treatment. Certain bacterial genera/species, such as *Akkermansia* [NSCLC, RCC (Routy et al., 2018; Zheng et al., 2019), and hepatocellular carcinoma (Zheng et al., 2019), *Clostridiales* [melanoma; (Zheng et al., 2019)] and *Alistipes putredinis* [NSCLC; (Jin et al., 2019)] have been found to be enriched in patients with favorable clinical outcomes. Recent studies demonstrate that fecal microbiota transplantation (FMT) modulated the tumor microbiome and affected tumor growth as well as tumor immune infiltration in a PDAC mice model (Thomas et al., 2018; Riquelme et al., 2019). This offers a therapeutic opportunity to manipulate the microbiome and potentially improve the life expectancy of PDAC patients with limited treatment options. However, the comprehensive understanding of bacteria clinically beneficial to ICI therapy remains incomplete, particularly in the context of pancreatic cancer. Further exploration is needed to identify and leverage specific bacteria that can optimize the efficacy of anti-PD-1/PD-L1 treatment in pancreatic cancer as explored in this article.

Therefore, the current study investigates the status of microbiota harbored by PDAC tissues. It demonstrates how the tumor tissue-harbored bacteria invaded potentially from other locations and their possible transmission to understand the effect of tissue microbiome on disease development. The study is focused on investigating the significance of tissue-associated microbiota, which could reflect the tumor state, topology, and overall tumor microenvironmental influence on the disease. These data reveal the complex relationship between individual tumor-inhabited microbial colonization that may help to develop strategies to improve patient outcomes. Therefore, we have investigated the enrichment of microbes highly localized at the tumor site. This is important to understand basic pathogenetic mechanisms that are driven by tissue-adhering bacteria in the progression from normal to PDAC, affecting the topology of pancreatic tissues to attain malignancy.

## 2 Methods

### 2.1 Patient sample collection

Archived pancreatic ductal adenocarcinoma tissues along with matched adjacent normal tissue samples were collected from Baptist Memorial Hospital, Memphis, TN, after appropriate institutional IRB approval and informed consent. Before being received at the research laboratory, the tissues were deidentified to block all patient personal information (Supplementary Table S1).

### 2.2 Sampling condition and processing for DNA extraction

Upon surgical resection, both pancreatic cancer and matched adjacent normal tissue samples were immediately placed in a sterile vial on dry ice until transferred to −80°C for DNA extraction. The samples were homogenized in sterile PBS using a homogenizer at 28,000 rpm. Considering the weight of the tissue, the final concentration of tissue in the PBS was 0.4 g/mL. DNA extraction from all the specimens was performed using the DNA extraction kit (MoBio Laboratories Inc.), following the manufacturer’s instructions. In brief, tissue samples were added to a bead beating tube for rapid and thorough homogenization. Cell lysis was performed by mechanical and chemical methods. Total genomic DNA was captured on a silica membrane in a spin column format. DNA was washed and eluted. This was followed by PCR analysis and other downstream applications. Most importantly, microbial DNA was purified from human tissue and enriched using the NEBNext Microbiome DNA Enrichment Kit, following the manufacturer’s instructions. In brief, genomic DNA, free proteins, proteinase A, SDS, and organic solvents were incubated for 10 min at room temperature, followed by washing beads two times in a bind/wash reaction buffer. Furthermore, MBD2-Fc magnetic beads were incubated with the reaction for 15 min at room temperature, and the supernatant containing enriched microbial DNA was collected. This method leads to the enrichment of microbial DNA from samples containing methylated host DNA (including human) by selective binding and removal of the CpG-methylated host DNA. Methylation at CpG sites in microbial species is rare, leaving the non-CpG-methylated (microbial) DNA in the supernatant. The quality and quantity of the extracted DNA were assessed by 1.0% agarose gel and NanoDrop (Thermo Scientific, United States), respectively.

### 2.3 Library preparation and sequencing

16S rRNA sequencing was performed following the method described by our group (Mukherjee et al., 2014, 2016) with minor modifications. In brief, library preparation was performed using the NEBNext Ultra™ DNA Library Prep Kit for Illumina sequencing (New England Biolabs), following the manufacturer’s recommendations. PCR was performed by thermocycling: 5 min at 94°C for initialization; 28 cycles of 3 min denaturation at 94°C, 40 s annealing at 53°C, and 1 min extension at 72°C; followed by 5 min final elongation at 72°C. We used three replicates per sample, and each PCR product of the same sample was mixed. The amplicon products from different samples were purified using Agencourt Ampure beads (Agencourt Bioscience Corporation, Beverly, MA, USA). The library quality was assessed on a Qubit@ 2.0 Fluorometer (Thermo Scientific) and Agilent Bioanalyzer 2,100 system. Finally, the library was sequenced on an Illumina Hiseq 2,500 platform, and 250-bp paired-end reads were generated.

### 2.4 Bioinformatics analysis

Raw read files in FASTQ format obtained from the sequencing platform were assessed for quality using FastQC v0.12.1 (Andrews, 2010), followed by trimming by Trimmomatic V0.33 (Bolger et al., 2014), to remove low-quality bases and adapter sequences to improve data quality. Paired-end clean reads were merged using FLASH V1.2.11, based on some specific criteria described elsewhere (Magoč and Salzberg, 2011). The microbial taxonomy analysis was performed on QIIME2 v2019.10 (Caporaso et al., 2010). In brief, DADA2 (Callahan et al., 2016) was employed for denoising and removing chimeric sequences. After the removal of singleton sequences, operational taxonomic unit (OTU) picking was performed at 3% divergence (97% similarity) (Dowd et al., 2008a,b; Edgar, 2010; Swanson et al., 2011). Taxonomic classification of OTUs was carried out using BLASTn against a curated SILVA SSU database v 138.1. The results of the taxonomic classification were used to generate the OTU file and associated taxonomy file for further downstream analysis (otu_count_ Supplementary Table S2, Sequences_otu_Supplementary Table S3, and Taxonomy_ Supplementary Table S4). The microbial community and diversity analysis was performed and visualized using different R packages (phyloseq, ggplot, vegan, and tidyverse), MicrobiomeAnalyst server (Dhariwal et al., 2017), and SHAMAN (Volant et al., 2020).

### 2.5 Expression of PD-L1 using qRT-PCR

RNA extraction from fresh tissues was performed using the RNeasy Midi Kit (catalog number 75144, Qiagen). To assess the relative expression levels of PD-L1 mRNA, a quantitative reverse transcription polymerase chain reaction (RT-PCR) was conducted. This involved the use of sequence-specific primers for human PD-L1: forward primer: 5′- CCA AGG CGC AGA TCA AAG AGA −3′ and reverse primer: 5′- AGG ACC CAG ACT AGC AGC A − 3′. These primers were utilized to amplify and analyze the expression of the PD-L1 gene. The relative expression of the PD-L1 gene was normalized by a housekeeping gene (GAPDH) and expressed as 2^^-(ΔΔCt)^ (Crucello et al., 2019).

### 2.6 Statistical analyses

The statistical significance of the abundances of phyla and genera between the groups has been tested using the Mann–Whitney method at a value of *p* level of <0.05. A PREANOVA NMDS (non-metric multidimensional scaling) plot was generated in the R platform using the ggplot2 package to visualize the data. The stress value and value of p indicate how well the data fit the NMDS model, and the statistical significance of the differences is observed. Furthermore, the analysis of the linear discriminant analysis (LDA) scores for differential genera between the two groups (normal and tumor) was conducted using LEfSe (Chang et al., 2022) in MicrobiomeAnalyst. All the experiments were performed in triplicate to minimize the impact of variability and increase confidence in the observed findings.

## 3 Results

### 3.1 Clinical characteristics of the cohort

This cohort comprises patients with diverse clinical characteristics, including varying ages, genders, and racial backgrounds (Supplementary Table S1). Individuals receive a diagnosis of PDAC, exhibiting varying tumor sizes, nodal engagement, and stages. Histological features range from well-differentiated to poorly-differentiated tumors, indicating a spectrum of disease severity and prognosis within this cohort and highlighting the heterogeneity of this patient population.

### 3.2 Taxonomic composition and abundances of bacterial phyla in pancreatic cancer

**Figure 1:**
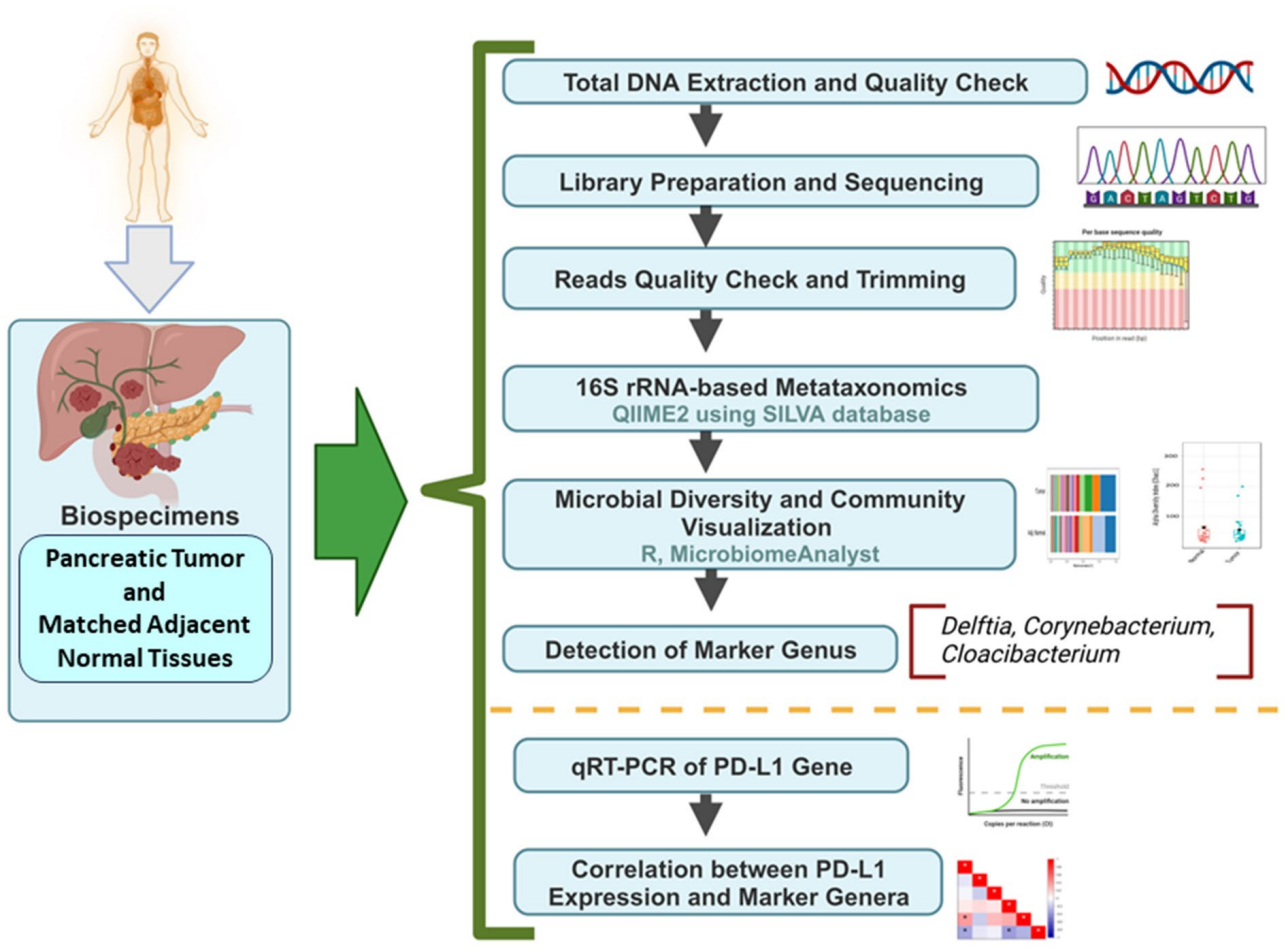
The detailed workflow from sample collection to downstream bioinformatics analysis.

**Figure 2:**
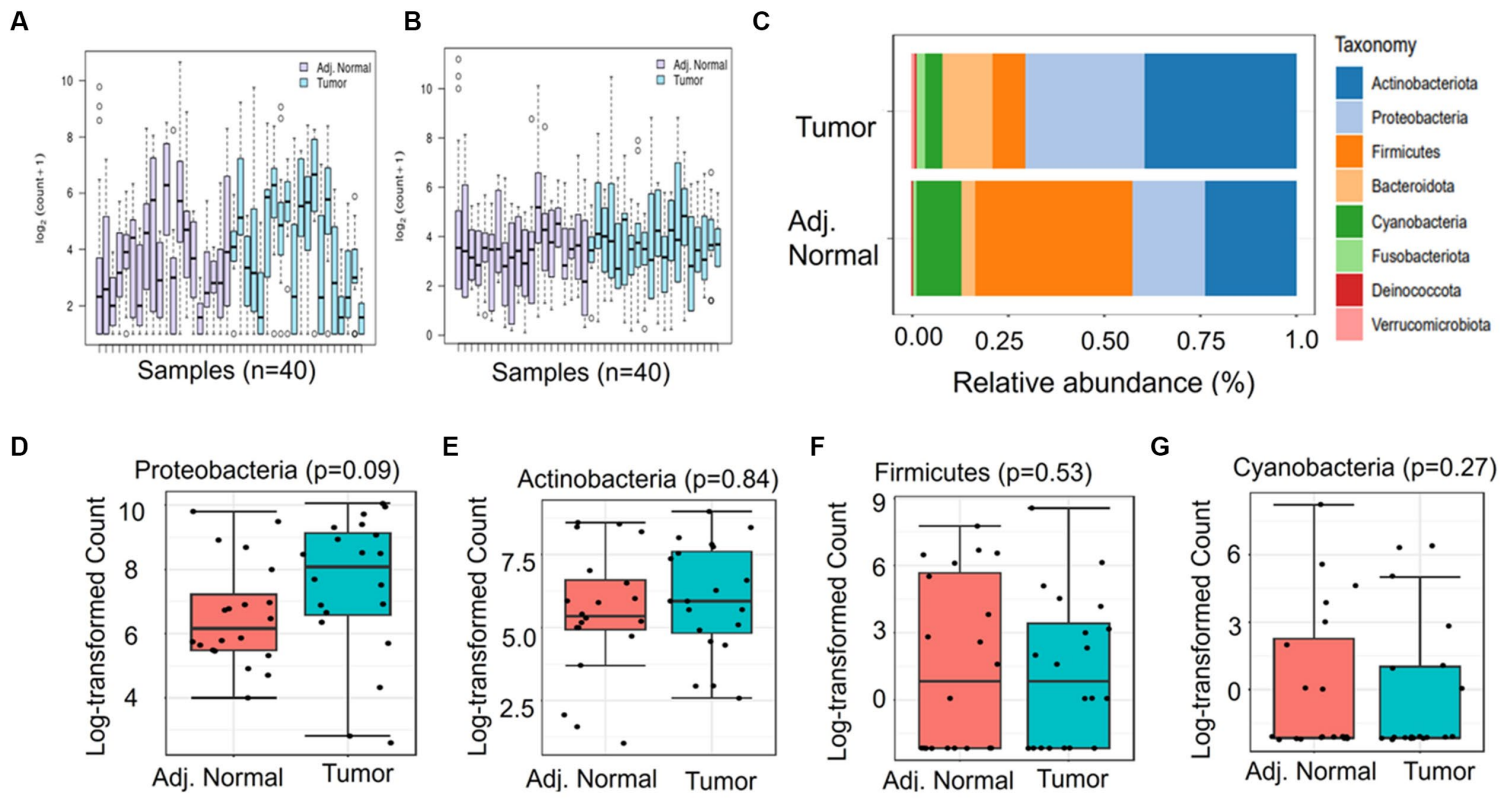
Differential abundance of phyla in adjacent normal and tumor tissues. **(A)** and **(B)** represent the log OTU count before and after normalization, respectively. **(C)** indicates the relative abundance of major phyla in adjacent normal and tumor tissues. **(D-G)** The Mann–Whitney significance test of the differential abundance of phyla between the adjacent normal and tumor groups.

DNA was extracted from freshly collected pancreatic tumor samples and matched adjacent normal samples, followed by sequencing and downstream bioinformatics analysis (Figure 1). The abundances of bacterial phyla have been assessed in tumor and adjacent normal tissues and presented in Figure 2. Due to differences in sequence depth, normalization is recommended before microbiome data analysis. The log-transformed OTU count before and after the normalization is presented in Figures 2A,B, respectively. The result indicates that five phyla, namely, Proteobacteria, Actinobacteroita, Firmicutes, Bacteroidota, and Cyanobacteria, are the most important contributors in these two tissue types (Figure 2C); however, their relative abundances vary between these two groups. For example, the abundance of Firmicutes (41.1%) was recorded to be very high in adjacent normal tissues, followed by Actinobacteroita (23.7%), Proteobacteria (18.7%), and Cyanobacteria (11.6%). Similarly, in tumor tissues, the abundances of Actinobacteroita (39.2%), Proteobacteria (31.3%), and Bacteroidota (14.4%) were high. However, the differential abundances of these phyla between groups (tumor and adjacent normal tissues) were not significant at a value of *p* level of <0.05 (Figures 2D–G).

### 3.3 Taxonomic composition indicates distinct bacterial genera found in pancreatic cancer

**Figure 3:**
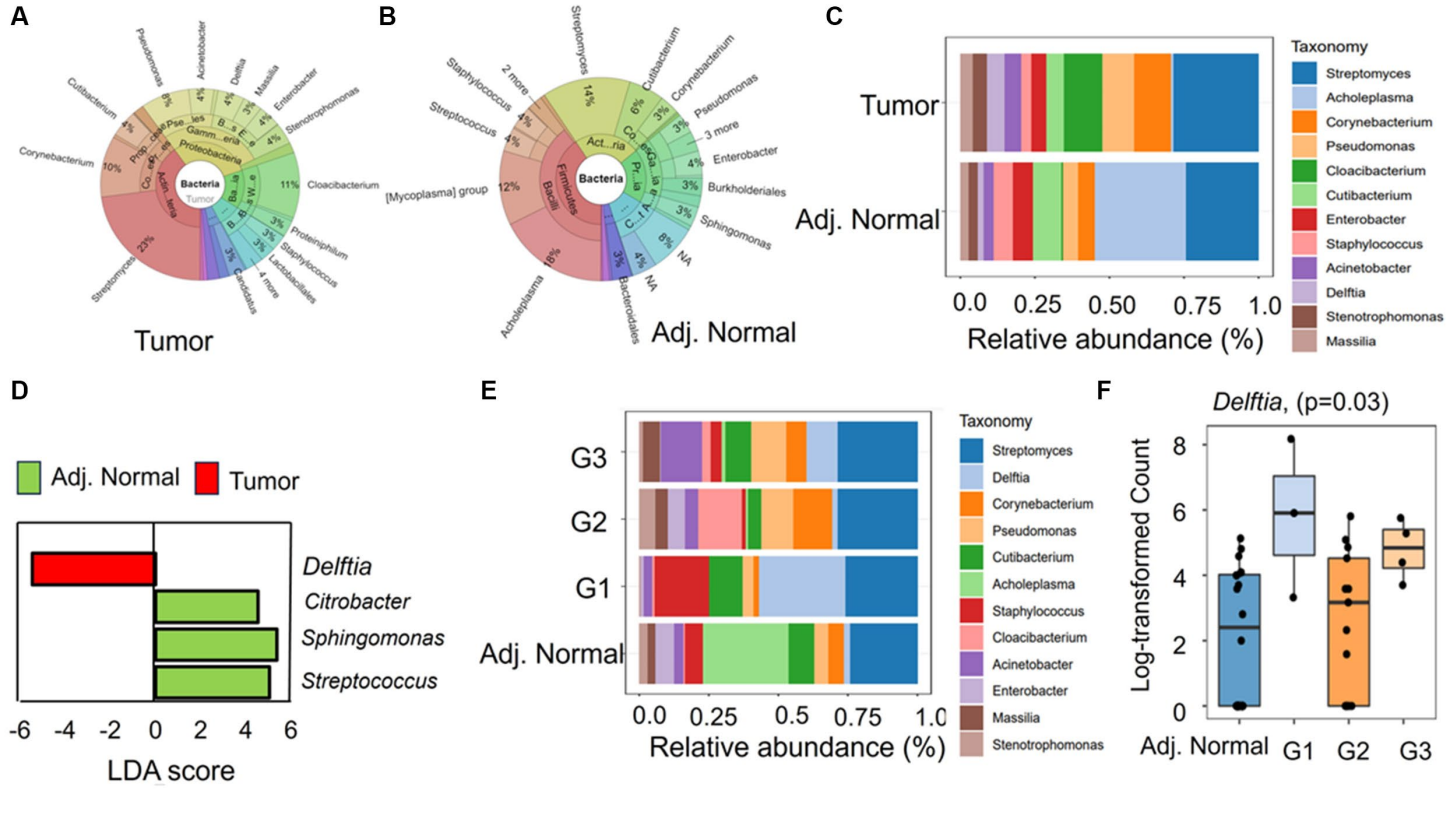
The taxonomic composition and differential abundance of genus between the groups. **(A,B)** Krona chart representing the percent abundances of genera in tumor and adjacent normal groups, respectively. **(C)** The differential relative abundance of the most dominant genera between the groups. **(D)** Linear discriminant analysis (LDA) effect size (LEfSe) performed on the microbial genus community abundance at a value of *p* level of <0.05. The candidates with significant LDA scores were represented in the graph. **(E)** The relative abundance of the genus in different tumor differentiation stages (G1, G2, and G3) along with adjacent. Normal tissues. **(F)** Mann–Whitney significance test of *Delftia* between different tumor stage groups and adjacent normal groups.

The genus abundances in both the tumor and adjacent normal groups are visually represented using a Krona graph in Figure 3A,B, respectively (Supplementary Figure S1). In the tumor group (Figure 3A), a higher prevalence of bacterial genera such as *Streptomyces*, *Cloacibacterium*, and *Corynebacterium* was observed. Conversely, in the adjacent normal tissues (Figure 3B), the genus *Acholeplasma* was the most predominant, followed by *Streptomyces*.

At the genus level, the differing taxonomic profiles between tumor and adjacent normal tissues revealed specific genera being exclusively present in tumor tissues, such as *Candidatus_Obscuribacter* (prevalence 10%) and *Nocardioides* (prevalence 10%). In contrast, two bacterial genera, such as *Atopostipes* (prevalence 15%) and *Veillonella* (prevalence 10%), were exclusively present in adjacent normal tissues. The most prevalent genera in tumor samples were *Streptomyces* (prevalence 100% and relative abundance 28.4%), *Cutibacterium* (prevalence 100% and relative abundance 12.9%), and *Pseudomonas* (prevalence 90% and relative abundance 10.7%) (Figure 3C). In contrast, *Cutibacterium* (prevalence 100% and relative abundance 9.5%), *Pseudomonas* (prevalence 100% and relative abundance 5%), and *Streptomyces* (prevalence 90% and relative abundance 23.4) were prevalent in adjacent normal tissues (Figure 3C). Interestingly, in the adjacent normal tissues, the genus *Acholeplasma* had a notably high relative abundance of 30.6%, while its prevalence in the samples was only 15% (Figure 3C). We further conducted LDA analysis to pinpoint significant (*p* < 0.05) features (genera), distinguishing these two groups. Intriguingly, three bacterial genera, namely, *Citrobacter*, *Sphingomonas*, and *Streptococcus,* displayed positive LDA scores (4.53, 5.37, and 5.05, respectively) in the adjacent normal group compared with the tumor group (Figure 3D). However, the genus *Delftia* exhibited a negative LDA score (−5.46), signifying its higher abundance in tumor tissues compared with adjacent normal tissues (Figure 3D).

Furthermore, we aimed to explore connections between bacterial abundance and clinical characteristics. Our approach involved categorizing the tumor group into three segments based on differentiation (G1, G2, and G3). The variations in genus composition underscore the presence of differentially abundant genera across various tumor stages. For instance, *Gemella* (prevalence 7.69%), *Lactobacillus* (prevalence 7.69%), *Microlunatus* (prevalence 7.69%), and *Bacillus* (prevalence 15.38%) exclusively appeared in G3 stage samples. On the other hand, a few genera such as *Cetobacterium*, *Enterococcus*, and *Fusobacterium* were detected in a few samples of the G2 and G3 groups but completely absent in G1 stage samples. Interestingly, in the G1 stages, where tumors are well differentiated, *Delftia* and *Staphylococcus* displayed higher abundances compared with G2 (well to moderately differentiated) and G3/G4 (poorly differentiated). The relative abundance and prevalence of *Delftia* were 31.0 and 100%, respectively, in G1 samples, while *Cloacibacterium* (relative abundance 15.7% and prevalence 15.38%) and *Actinobacter* (relative abundance 14.9% and prevalence 66.6%) were abundant in G2 and G3, respectively (Figure 3E). The substantial abundance of *Delftia* in G1, particularly when compared with adjacent normal tissues, suggests its role in tumor initiation and development and characteristics of well-differentiated cells. The resurgence of *Delftia* in G3 might indicate heightened cellular activity in poorly differentiated tumors, demanding aggressive proliferation and alterations in pathways or the tumor microenvironment. Furthermore, the Mann–Whitney analysis emphasizes the significant difference in the abundance of *Delftia* between these groups (Figure 3F). These findings emphasize the unique and crucial role that *Delftia* may play in tumor development and progression, opening avenues for further exploration.

### 3.4 Bacterial diversity in normal and tumor tissues

**Figure 4:**
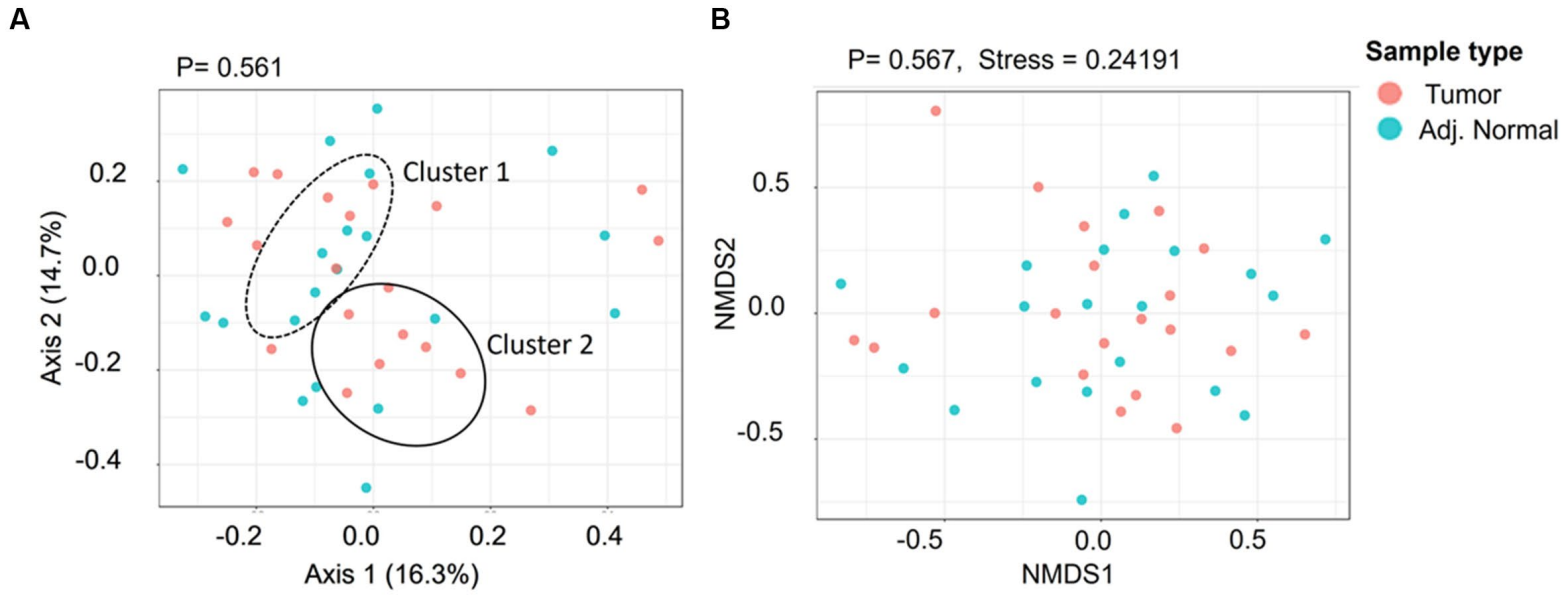
The genus level beta diversity between the tumor and adjacent normal groups. **(A)** Principal coordinates analysis (PCoA) indicates the genus-level taxonomic diversity between groups. Cluster 1 and Cluster 2 indicate the adjacent normal and tumor groups, respectively. **(B)** 7. Non-metric multidimensional scaling (NMDS) ordination plot presenting taxonomic composition diversity between the groups.

The alpha diversity of microbial community in both normal and tumor samples has been investigated using Shannon and Simpson diversity indices. The Shannon diversity index indicates the species diversity in a community and is recorded to be 1.78 and 1.77 for adjacent normal and tumor groups, respectively (Supplementary Figure S2). Similarly, the Simpson index of adjacent normal and tumor groups was recorded to be 0.77 and 0.78, respectively. However, the species diversity and their relative abundances between these two groups are not significant at *p* < 0.05. To understand the similarities or differences between the microbiome of the tumor and adjacent normal tissues, we performed beta diversity (PCoA and NMDS) analyses using Bray–Curtis dissimilarity distance (Figure 4). Beta diversity essentially allows the comparison of the community of bacteria considering both how many different taxa are in the sample and their phylogenetic relationship. The PCoA plot indicated two overlapping clusters (one for tumor tissue and one for adjacent tissue); however, the genus diversity was not significant at a value of *p* level of <0.05 (Figure 4A). Furthermore, the NMDS plot was generated to visualize the difference in taxonomic composition between tumor and adjacent normal groups. No significant difference was observed between these two groups (Figure 4B, Pre-ANOVA, R^2^ = 0.02, *p* = 0.56).

### 3.5 Correlation between PD-L1 expression in tumor tissues and dominant bacterial genera

**Figure 5:**
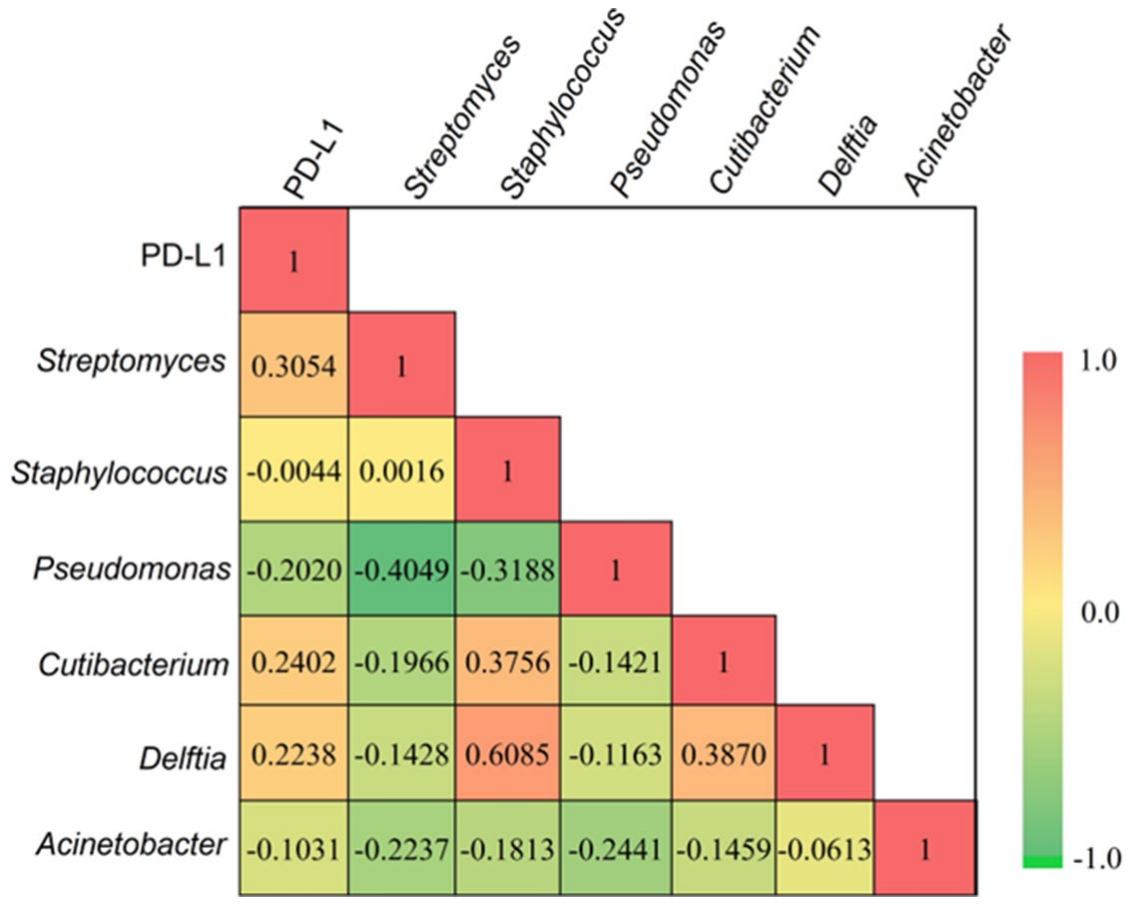
The multiple correlation coefficient (at *p* < 0.05) demostrates between the most prevalent bacterial genera and relative expression of PD-L1 in tumor tissues. Red and green gradients signify the positive and negative correlation, respectively.

The correlation of PD-L1 expression (Supplementary Table S5) in cancer lesions with the most prevalent genera (present in at least 60% of the sample) has been calculated using multiple correlation coefficient statistics (Figure 5**)**. The relative expressions of PD-L1 in tumor lesions were computed using qRT-PCR after normalizing the expressions in adjacent normal tissues. Our result depicted a positive correlation of *Streptomyces* (0.3054), *Cutibacterium* (0.2402), and *Delftia* (0.2238) with PD-L1 expression, while the correlation of *Pseudomonas* (−0.2020), *Staphylococcus* (−0.0044), and *Acinetobacter* (−0.1030) with PD-L1 was recorded to be negative. However, none of these interactions are significant at *p* < 0.05.

## 4 Discussion

The challenge of early PDAC detection persists, with the mechanisms underlying pancreatic cancer progression still not fully understood. Despite reported oncogenes, diagnosis and predicting outcomes remain challenging. While the role of microbiota in diagnosis and prognosis is recognized, its impact on the natural history of PDAC is insufficiently explored. This study investigates tissue-invaded bacteria, exploring their contribution to disease development, marking the first exploration of PDAC tumor microbiome at both genera and species levels, and providing valuable insights into pathogenesis.

Several metagenomic and transcriptomic studies resulted in the development of novel specific and sensitive biomarkers for the diagnosis of PDAC at the earliest stages. The current study presents 16S rRNA taxonomic profiling, revealing either overabundance or underabundance of distinct bacterial communities within the microbiota of pancreatic cancer tissues. Using surgically resected PDAC tissues and comparing them with matched adjacent normal tissues from the same patient, we were able to demonstrate distinct occurrences or abundances of bacterial taxa that harbor pancreatic tissue. Our findings provide evidence that the pancreatic tissue occupies substantial indices of bacterial diversity between-person variability in the relative abundances of bacterial taxa at the genera level in the pancreas. Our study demonstrates, for the first time in human PDAC patients, the distinct microbiota signatures colonizing pancreatic tumors and the matched adjacent normal tissue, and this colonization can modify the overall microbiome of the tumor. Interestingly, the phylum Bacteroidota (14.3%) was detected to be abundant in tumor tissues. The member of Bacteroidota belongs to different ecological habitats such as water, soil, ocean, and the gastrointestinal tract and consist of four major classes, namely, Flavobacteria, Cytophagia, Bacteroidia, and Sphingobacteria (Thomas et al., 2011). However, many bacterial candidates belonging to the class Bacteroidia are reported to the potential pathogens that cause different types of health complications (Wexler, 2007). The genus-level analysis showed high abundances of few important bacterial genera (such as *Corynebacterium*, *Delftia*, *Cloacibacterium*, and *Pseudomonas*) in cancer lesions compared with matched normal tissues from the same patient (Figure 3). *Corynebacterium* is a gram-positive bacterium, and many species of this genus have been reported to cause infections in humans and other animals (Oliveira et al., 2017). Even more, few previous studies have reported the close association of *Corynebacterium* with cancer patients and patients with solid tumors (Martins et al., 2009; Oresta et al., 2021). On the other hand, *Delftia* is a gram-negative bacterium and also have been reported to cause different types of infections in immunocompromised patients, including cancer (Bilgin et al., 2015; Brady et al., 2018). This indicates that these genera may contribute to risk stratification for pancreatic cancer. Many species of *Pseudomonas* (especially *P. aeruginosa*) are known to be one of the risk factors in the occupational environment, leading to respiratory disease. *P. aeruginosa*, due to its metabolite properties, contributes to nitric oxide (NO)-related carcinogenesis, such as oral cancer (Kakabadze et al., 2020) and NO signaling pathway as a pathogenic driver in pancreatic cancer (Wang et al., 2015; Fujita et al., 2019). However, at the genus level, the beta diversity (both PCoA and NMDS) does not show any significant differences at a value of p level of <0.05. Given the findings from this study, where two different tissue specimens were examined in the same subject and show difference in microbial inhabitation, it may also be plausible that everyone has a unique microbiome profile that exists in different gastrointestinal tissues, and that bacterial translocation from the gut to the pancreas might be occurring. Most bacterial communities found in the tumoral milieu are commonly present in the gut microbiome, but investigating the tumor region is more important to determine the relevance of particular microbial colonization. This represents a significant unmet need, as most chemotherapeutic and immunotherapeutic agents that have proven efficacy in other malignancies have limited efficacy in PDAC. Since the gut harbors numerous good and bad bacteria, pancreatic tumors present the microbiota that is directly associated with disease prevalence and progression.

Recent studies have shown that the microbiota affects the responses to immune checkpoint blockade therapy in patients with cancer (Roy and Trinchieri, 2017; Routy et al., 2018; Saus et al., 2019). The fecal microbiota transplantation is an innovative investigational treatment that has been reported to augment and restore the human immune system, leading to increased sensitivity to immunotherapy (Smits et al., 2013; Kang and Cai, 2021). Organ-specific microbiomes have been recently given more importance due to their role in cancer growth (Schwabe and Jobin, 2013). Data suggest that microbiota adjustment may present a novel strategy for improving the efficacy of immunotherapies for cancer, particularly checkpoint blockade approaches targeting the cytotoxic T-lymphocyte-associated protein 4 and programmed cell death ligand-1 (PD-L1) pathways (Goc and Sonnenberg, 2022; Park et al., 2022). Specifically, our analysis establishes a positive compelling link between PD-L1 expression in PDAC tumor tissues and bacterial genera *(Streptomyces* and *Delftia)*, suggesting a nuanced microbial influence on the tumor microenvironment. These insights not only contribute to our understanding of PDAC but also pave the way for targeted interventions aimed at harnessing the microbiota to optimize immunotherapeutic approaches in cancer, with particular emphasis on the PD-L1 pathway. This study positions microbiota modulation as a promising avenue for advancing precision medicine and personalized immunotherapy strategies in the realm of cancer treatment.

In summary, our results highlight the growing importance of microbiota in influencing responses to cancer treatments. We have explored the diversity of bacterial phyla and genera, delving into the intricacies of their abundances in the tumor and adjacent normal tissues. Hence, this study stands out by specifically examining microbes in pancreatic tissue, revealing unique microbial populations in both tumor and adjacent normal tissues, which is consistent with some studies demonstrating the dominance of the most prevalent species in each of them (Kartal et al., 2022; Tan et al., 2022). This contrasts with some previous studies that highlighted no differences in microbial populations between tumors and adjacent normal tissues (Nejman et al., 2020). Despite some differences, there are shared observations with the previous studies investigating pancreatic tissue microbiome, such as the prevalence of Proteobacteria and Firmicutes, indicating potential microbial involvement in pancreatic cancer development (Geller et al., 2017; Nejman et al., 2020). The current study extends this knowledge by providing a comprehensive analysis at the genera and species levels, emphasizing the unique microbial signatures in both tumor and adjacent normal tissues and offering insights into microbial signatures associated with different stages of tumor progression. Moreover, our findings highlight the influence of microbiota on immune checkpoint-associated proteins, revealing a positive correlation between increased PD-L1 expression and the presence of specific genera (*Streptomyces*, *Cutibacterium*, and *Delftia*). This direct positive association between *Streptomyces, Cutibacterium*, and *Delftia* and PD-L1 expression suggests a nuanced microbial influence on the tumor microenvironment, opening avenues for targeted interventions. Therefore, acknowledgment of organ-specific microbiomes, particularly in their contribution to cancer development, emphasizes the potential of microbiota modifications as an innovative approach to bolster the treatment, including immunotherapies.

Despite these advancements, our study acknowledges a limitation in its sample size, urging the need for a more expansive and diverse population for enhanced generalizability. Future research should prioritize extensive validation studies, collaborating across research centers and incorporating larger, diverse samples. Additionally, understanding the molecular underpinnings of PDAC through our results holds the potential for stratifying patients for targeted therapies, ultimately improving treatment outcomes and patient care. By addressing these limitations and pursuing these future directions, we strive to propel PDAC research forward and translate our findings into impactful clinical applications. Altogether, this study unveils crucial insights into the pancreatic tumor tissue microbiome, showcasing distinct microbial communities that can impact PDAC pathogenesis and prognosis. Notably, our study lays the groundwork by uncovering distinct microbial colonization patterns in both PDAC tumors and adjacent normal tissues at the broader compositional level. This opens promising avenues for leveraging the microbiome in PDAC diagnostics and therapeutics.

## 5 Conclusion

Overall, this study provides an important insight into pancreatic tumor tissue microbiome at both phylum and genus levels. The findings reveal distinct microbial communities within pancreatic tumor lesions that potentially can influence PDAC pathogenesis and prognosis of patients. Notably, the high abundance of *Delftia* in tumors emerges as a potential molecular signature for disease diagnosis. This study also highlights the influence of microbiota on the expression of immune checkpoint-associated proteins (such as PD-L1, which is involved in immune checkpoint blockade therapy) as we observed a positive correlation between increased PD-L1 expression and the presence of *Streptomyces*, *Cutibacterium*, and *Delftia.* These findings open new avenues for exploiting the microbiome as a potential diagnostic and therapeutic target in PDAC. However, further understanding regarding the relevance of specific microbial colonization in the tumors will be highly useful for improving the therapy/management of pancreatic cancer patients; thus, future studies are warranted in this important area to address the urgent need for developing more effective diagnostic/therapeutic modalities for this devastating cancer.

## Data availability statement

The datasets presented in this study can be found in online repositories. The names of the repository/repositories and accession number(s) can be found in the article/Supplementary material. This manuscript contains metagenomic sequence data from tumor tissues (*n* = 20) and adjacent tissues (*n* = 20). The metataxonomic sequence datasets associated with this paper are archived at NCBI Sequence Read Archive (SRA) under the BioProject accession number PRJNA879535.

## Ethics statement

The studies involving humans were approved by Baptist memorial Hospital Institutional Review board. The studies were conducted in accordance with the local legislation and institutional requirements. The participants provided their written informed consent to participate in this study.

## Author contributions

SK: Conceptualization, Data curation, Formal analysis, Funding acquisition, Investigation, Methodology, Project administration, Resources, Software, Supervision, Validation, Visualization, Writing – original draft, Writing – review & editing. GB: Conceptualization, Investigation, Validation, Writing – review & editing, Data curation, Methodology, Software. SS: Investigation, Methodology, Writing – review & editing. DJ: Data curation, Formal analysis, Investigation, Writing – review & editing. BC: Investigation, Methodology, Visualization, Writing – review & editing. AD: Investigation, Methodology, Software, Writing – review & editing. PB: Conceptualization, Data curation, Investigation, Methodology, Project administration, Supervision, Validation, Visualization, Writing – review & editing. MY: Investigation, Validation, Visualization, Writing – review & editing. SB: Conceptualization, Investigation, Project administration, Resources, Supervision, Validation, Visualization, Writing – review & editing. SC: Validation, Writing – review & editing, Funding acquisition, Project administration, Supervision, Visualization, Conceptualization, Investigation.

## References

[ref1] Almeida J. Galhenage S. Yu J. Kurtovic J. Riordan S. M. (2006). Gut flora and bacterial translocation in chronic liver disease. World J. Gastroenterol. 12, 1493–1502. doi: 10.3748/wjg.v12.i10.1493, PMID: 16570339 PMC4124279

[ref2] Andrews S. FastQC: a quality control tool for high throughput sequence data Available at: [https://www.bioinformatics.babraham.ac.uk/projects/fastqc/]. (2010).

[ref3] Bilgin H. Sarmis A. Tigen E. Soyletir G. Mulazimoglu L. (2015). *Delftia acidovorans*: a rare pathogen in immunocompetent and immunocompromised patients. Canadian J Infectious Dis Med Microbiol 26, 277–279. doi: 10.1155/2015/973284, PMID: 26600818 PMC4644013

[ref4] Bolger A. M. Lohse M. Usadel B. (2014). Trimmomatic: a flexible trimmer for Illumina sequence data. Bioinformatics 30, 2114–2120. doi: 10.1093/bioinformatics/btu170, PMID: 24695404 PMC4103590

[ref5] Borghaei H. Paz-Ares L. Horn L. Spigel D. R. Steins M. Ready N. E.. (2015). Nivolumab versus docetaxel in advanced nonsquamous non–small-cell lung Cancer. N. Engl. J. Med. 373, 1627–1639. doi: 10.1056/NEJMoa1507643, PMID: 26412456 PMC5705936

[ref6] Brady M. T. Leber A. Less S. S. (2018). “Less commonly encountered nonenteric gram-negative bacilli,” in Principles and practice of pediatric infectious diseases. eds. Prober C. G. Fischer M. (Fifth Edution, Philadelphia: Elsevier), 855–859e.

[ref7] Bultman S. J. (2016). The microbiome and its potential as a cancer preventive intervention. Semin. Oncol. 43, 97–106. doi: 10.1053/j.seminoncol.2015.09.001, PMID: 26970128 PMC4789109

[ref8] Callahan B. J. McMurdie P. J. Rosen M. J. Han A. W. Johnson A. J. A. Holmes S. P. (2016). DADA2: high-resolution sample inference from Illumina amplicon data. Nat. Methods 13, 581–583. doi: 10.1038/nmeth.3869, PMID: 27214047 PMC4927377

[ref9] Caporaso J. G. Kuczynski J. Stombaugh J. Bittinger K. Bushman F. D. Costello E. K.. (2010). QIIME allows analysis of high-throughput community sequencing data. Nat. Methods 7, 335–336. doi: 10.1038/nmeth.f.303, PMID: 20383131 PMC3156573

[ref10] Chang F. He S. Dang C. (2022). Assisted selection of biomarkers by linear discriminant analysis effect size (LEfSe) in microbiome data. JoVE Visualized Experiments:e61715. doi: 10.3791/61715-v, PMID: 35635468

[ref11] Chaput N. Lepage P. Coutzac C. Soularue E. Le Roux K. Monot C.. (2017). Baseline gut microbiota predicts clinical response and colitis in metastatic melanoma patients treated with ipilimumab. Ann. Oncol. 28, 1368–1379. doi: 10.1093/annonc/mdx108, PMID: 28368458

[ref12] Crucello A. Furtado M. M. Chaves M. D. Sant’Ana A. S. (2019). Transcriptome sequencing reveals genes and adaptation pathways in *Salmonella Typhimurium* inoculated in four low water activity foods. Food Microbiol. 82, 426–435. doi: 10.1016/j.fm.2019.03.016, PMID: 31027802

[ref13] Dal Molin M. Zhang M. de Wilde R. F. Ottenhof N. A. Rezaee N. Wolfgang C. L.. (2015). Very long-term survival following resection for pancreatic Cancer is not explained by commonly mutated genes: results of whole-exome sequencing analysis. Clin. Cancer Res. 21, 1944–1950. doi: 10.1158/1078-0432.CCR-14-2600, PMID: 25623214 PMC4401626

[ref14] Davenport E. R. Cusanovich D. A. Michelini K. Barreiro L. B. Ober C. Gilad Y. (2015). Genome-wide association studies of the human gut microbiota. PLoS One 10:e0140301. doi: 10.1371/journal.pone.0140301, PMID: 26528553 PMC4631601

[ref15] David L. A. Maurice C. F. Carmody R. N. Gootenberg D. B. Button J. E. Wolfe B. E.. (2014). Diet rapidly and reproducibly alters the human gut microbiome. Nature 505, 559–563. doi: 10.1038/nature12820, PMID: 24336217 PMC3957428

[ref16] Del Castillo E. Meier R. Chung M. Koestler D. C. Chen T. Paster B. J.. (2019). The microbiomes of pancreatic and duodenum tissue overlap and are highly subject specific but differ between pancreatic Cancer and noncancer subjects. Cancer Epidemiol. Biomark. Prev. 28, 370–383. doi: 10.1158/1055-9965.EPI-18-0542, PMID: 30373903 PMC6363867

[ref17] Dhariwal A. Chong J. Habib S. King I. L. Agellon L. B. Xia J. (2017). MicrobiomeAnalyst: a web-based tool for comprehensive statistical, visual and meta-analysis of microbiome data. Nucleic Acids Res. 45, W180–W188. doi: 10.1093/nar/gkx295, PMID: 28449106 PMC5570177

[ref18] Dowd S. E. Callaway T. R. Wolcott R. D. Sun Y. McKeehan T. Hagevoort R. G.. (2008a). Evaluation of the bacterial diversity in the feces of cattle using 16S rDNA bacterial tag-encoded FLX amplicon pyrosequencing (bTEFAP). BMC Microbiol. 8:125–132. doi: 10.1186/1471-2180-8-125, PMID: 18652685 PMC2515157

[ref19] Dowd S. E. Sun Y. Wolcott R. D. Domingo A. Carroll J. A. (2008b). Bacterial tag-encoded FLX amplicon pyrosequencing (bTEFAP) for microbiome studies: bacterial diversity in the ileum of newly weaned Salmonella-infected pigs. Foodborne Pathog. Dis. 5, 459–472. doi: 10.1089/fpd.2008.0107, PMID: 18713063

[ref20] Edgar R. C. (2010). Search and clustering orders of magnitude faster than BLAST. Bioinformatics 26, 2460–2461. doi: 10.1093/bioinformatics/btq461, PMID: 20709691

[ref21] Fierer N. Hamady M. Lauber C. L. Knight R. (2008). The influence of sex, handedness, and washing on the diversity of hand surface bacteria. Proc. Natl. Acad. Sci. 105, 17994–17999. doi: 10.1073/pnas.0807920105, PMID: 19004758 PMC2584711

[ref22] Fujita M. Somasundaram V. Basudhar D. Cheng R. Y. S. Ridnour L. A. Higuchi H.. (2019). Role of nitric oxide in pancreatic cancer cells exhibiting the invasive phenotype. Redox Biol. 22:101158. doi: 10.1016/j.redox.2019.101158, PMID: 30852389 PMC6409427

[ref23] Geller L. T. Barzily-Rokni M. Danino T. Jonas O. H. Shental N. Nejman D.. (2017). Potential role of intratumor bacteria in mediating tumor resistance to the chemotherapeutic drug gemcitabine. Science 357, 1156–1160. doi: 10.1126/science.aah5043, PMID: 28912244 PMC5727343

[ref24] Goc J. Sonnenberg G. F. (2022). Harnessing microbiota to improve immunotherapy for gastrointestinal cancers. Cancer Immunol. Res. 10, 1292–1298. doi: 10.1158/2326-6066.CIR-22-0164, PMID: 36166399 PMC10424780

[ref25] Gopalakrishnan V. Spencer C. N. Nezi L. Reuben A. Andrews M. C. Karpinets T. V.. (2018). Gut microbiome modulates response to anti–PD-1 immunotherapy in melanoma patients. Science 359, 97–103. doi: 10.1126/science.aan4236, PMID: 29097493 PMC5827966

[ref26] Hargadon K. M. Johnson C. E. Williams C. J. (2018). Immune checkpoint blockade therapy for cancer: an overview of FDA-approved immune checkpoint inhibitors. Int. Immunopharmacol. 62, 29–39. doi: 10.1016/j.intimp.2018.06.001, PMID: 29990692

[ref27] Huse S. M. Ye Y. Zhou Y. Fodor A. A. (2012). A core human microbiome as viewed through 16S rRNA sequence clusters. PLoS One 7:e34242. doi: 10.1371/journal.pone.0034242, PMID: 22719824 PMC3374614

[ref28] Jin Y. Dong H. Xia L. Yang Y. Zhu Y. Shen Y.. (2019). The diversity of gut microbiome is associated with favorable responses to anti–programmed death 1 immunotherapy in Chinese patients with NSCLC. J. Thorac. Oncol. 14, 1378–1389. doi: 10.1016/j.jtho.2019.04.007, PMID: 31026576

[ref29] Kakabadze M. Z. Paresishvili T. Karalashvili L. Chakhunashvili D. Kakabadze Z. (2020). Oral microbiota and oral cancer: review. Oncol. Rev. 14:476. doi: 10.4081/oncol.2020.476, PMID: 32676172 PMC7358985

[ref30] Kang Y. B. Cai Y. (2021). Faecal microbiota transplantation enhances efficacy of immune checkpoint inhibitors therapy against cancer. World J. Gastroenterol. 27, 5362–5375. doi: 10.3748/wjg.v27.i32.5362, PMID: 34539138 PMC8409158

[ref31] Kartal E. Schmidt T. S. B. Molina-Montes E. Rodríguez-Perales S. Wirbel J. Maistrenko O. M.. (2022). A faecal microbiota signature with high specificity for pancreatic cancer. Gut 71, 1359–1372. doi: 10.1136/gutjnl-2021-324755, PMID: 35260444 PMC9185815

[ref32] Kau A. L. Ahern P. P. Griffin N. W. Goodman A. L. Gordon J. I. (2011). Human nutrition, the gut microbiome and the immune system. Nature 474, 327–336. doi: 10.1038/nature10213, PMID: 21677749 PMC3298082

[ref33] Kostic A. D. Gevers D. Pedamallu C. S. Michaud M. Duke F. Earl A. M.. (2012). Genomic analysis identifies association of Fusobacterium with colorectal carcinoma. Genome Res. 22, 292–298. doi: 10.1101/gr.126573.111, PMID: 22009990 PMC3266036

[ref34] Ley R. E. (2010). Obesity and the human microbiome. Curr. Opin. Gastroenterol. 26, 5–11. doi: 10.1097/MOG.0b013e328333d751, PMID: 19901833

[ref35] Liu X.-b. Gao Z.-y. Sun C.-t. Wen H. Gao B. Li S.-b.. (2019). The potential role of P.Gingivalis in gastrointestinal cancer: a mini review. Infectious Agents and Cancer 14:23. doi: 10.1186/s13027-019-0239-4 31516546 PMC6734237

[ref36] Magoč T. Salzberg S. L. (2011). FLASH: fast length adjustment of short reads to improve genome assemblies. Bioinformatics 27, 2957–2963. doi: 10.1093/bioinformatics/btr507, PMID: 21903629 PMC3198573

[ref37] Martins C. S. Faria L. Souza M. Camello T. Velasco E. Hirata R. Jr.. (2009). Microbiological and host features associated with corynebacteriosis in cancer patients: a five-year study. Mem. Inst. Oswaldo Cruz 104, 905–913. doi: 10.1590/S0074-02762009000600015, PMID: 19876565

[ref38] Matson V. Fessler J. Bao R. Chongsuwat T. Zha Y. Alegre M.-L.. (2018). The commensal microbiome is associated with anti–PD-1 efficacy in metastatic melanoma patients. Science 359, 104–108. doi: 10.1126/science.aao3290, PMID: 29302014 PMC6707353

[ref39] Mitsuhashi K. Nosho K. Sukawa Y. Matsunaga Y. Ito M. Kurihara H.. (2015). Association of Fusobacterium species in pancreatic cancer tissues with molecular features and prognosis. Oncotarget 6, 7209–7220. doi: 10.18632/oncotarget.3109, PMID: 25797243 PMC4466679

[ref40] Motzer R. J. Escudier B. McDermott D. F. George S. Hammers H. J. Srinivas S.. (2015). Nivolumab versus Everolimus in advanced renal-cell carcinoma. N. Engl. J. Med. 373, 1803–1813. doi: 10.1056/NEJMoa1510665, PMID: 26406148 PMC5719487

[ref41] Muegge B. D. Kuczynski J. Knights D. Clemente J. C. González A. Fontana L.. (2011). Diet drives convergence in gut microbiome functions across mammalian phylogeny and within humans. Science 332, 970–974. doi: 10.1126/science.1198719, PMID: 21596990 PMC3303602

[ref42] Mukherjee N. Bartelli D. Patra C. Chauhan B. V. Dowd S. E. Banerjee P. (2016). Microbial diversity of source and point-of-use water in rural Haiti - a pyrosequencing-based metagenomic survey. PLoS One 11:e0167353. doi: 10.1371/journal.pone.0167353, PMID: 27936055 PMC5147895

[ref43] Mukherjee N. Dowd S. E. Wise A. Kedia S. Vohra V. Banerjee P. (2014). Diversity of bacterial communities of fitness center surfaces in a U.S. metropolitan area. Int. J. Environ. Res. Public Health 11, 12544–12561. doi: 10.3390/ijerph111212544, PMID: 25479039 PMC4276630

[ref44] Nejman D. Livyatan I. Fuks G. Gavert N. Zwang Y. Geller L. T.. (2020). The human tumor microbiome is composed of tumor type–specific intracellular bacteria. Science 368, 973–980. doi: 10.1126/science.aay9189, PMID: 32467386 PMC7757858

[ref45] Oliveira A. Oliveira L. C. Aburjaile F. Benevides L. Tiwari S. Jamal S. B.. (2017). Insight of genus Corynebacterium: ascertaining the role of pathogenic and non-pathogenic species. Front. Microbiol. 8:1937. doi: 10.3389/fmicb.2017.01937 29075239 PMC5643470

[ref46] Oresta B. Braga D. Lazzeri M. Frego N. Saita A. Faccani C.. (2021). The microbiome of catheter collected urine in males with bladder Cancer according to disease stage. J. Urol. 205, 86–93. doi: 10.1097/JU.0000000000001336, PMID: 32856979

[ref47] Park E. M. Chelvanambi M. Bhutiani N. Kroemer G. Zitvogel L. Wargo J. A. (2022). Targeting the gut and tumor microbiota in cancer. Nat. Med. 28, 690–703. doi: 10.1038/s41591-022-01779-2, PMID: 35440726

[ref48] Pushalkar S. Hundeyin M. Daley D. Zambirinis C. P. Kurz E. Mishra A.. (2018). The pancreatic Cancer microbiome promotes oncogenesis by induction of innate and adaptive immune suppression. Cancer Discov. 8, 403–416. doi: 10.1158/2159-8290.CD-17-1134, PMID: 29567829 PMC6225783

[ref49] Qin J. Li R. Raes J. Arumugam M. Burgdorf K. S. Manichanh C.. (2010). A human gut microbial gene catalogue established by metagenomic sequencing. Nature 464, 59–65. doi: 10.1038/nature08821, PMID: 20203603 PMC3779803

[ref50] Riquelme E. Zhang Y. Zhang L. Montiel M. Zoltan M. Dong W.. (2019). Tumor microbiome diversity and composition influence pancreatic Cancer outcomes. Cells 178, 795–806.e12. doi: 10.1016/j.cell.2019.07.008, PMID: 31398337 PMC7288240

[ref51] Rogers M. B. Aveson V. Firek B. Yeh A. Brooks B. Brower-Sinning R.. (2017). Disturbances of the perioperative microbiome across multiple body sites in patients undergoing Pancreaticoduodenectomy. Pancreas 46, 260–267. doi: 10.1097/MPA.0000000000000726, PMID: 27846140 PMC5235958

[ref52] Routy B. Chatelier E. L. Derosa L. Duong C. P. M. Alou M. T. Daillère R.. (2018). Gut microbiome influences efficacy of PD-1–based immunotherapy against epithelial tumors. Science 359, 91–97. doi: 10.1126/science.aan3706, PMID: 29097494

[ref53] Roy S. Trinchieri G. (2017). Microbiota: a key orchestrator of cancer therapy. Nat. Rev. Cancer 17, 271–285. doi: 10.1038/nrc.2017.13, PMID: 28303904

[ref54] Saus E. Iraola-Guzmán S. Willis J. R. Brunet-Vega A. Gabaldón T. (2019). Microbiome and colorectal cancer: roles in carcinogenesis and clinical potential. Mol. Asp. Med. 69, 93–106. doi: 10.1016/j.mam.2019.05.001, PMID: 31082399 PMC6856719

[ref55] Schwabe R. F. Jobin C. (2013). The microbiome and cancer. Nat. Rev. Cancer 13, 800–812. doi: 10.1038/nrc3610, PMID: 24132111 PMC3986062

[ref56] Sender R. Fuchs S. Milo R. (2016). Revised estimates for the number of human and Bacteria cells in the body. PLoS Biol. 14:e1002533. doi: 10.1371/journal.pbio.1002533, PMID: 27541692 PMC4991899

[ref57] Smits L. P. Bouter K. E. de Vos W. M. Borody T. J. Nieuwdorp M. (2013). Therapeutic potential of fecal microbiota transplantation. Gastroenterology 145, 946–953. doi: 10.1053/j.gastro.2013.08.058, PMID: 24018052

[ref58] Structure H. M. P. (2012). Function and diversity of the healthy human microbiome. Nature 486, 207–214. doi: 10.1038/nature11234 22699609 PMC3564958

[ref59] Swanson K. S. Dowd S. E. Suchodolski J. S. Middelbos I. S. Vester B. M. Barry K. A.. (2011). Phylogenetic and gene-centric metagenomics of the canine intestinal microbiome reveals similarities with humans and mice. ISME J. 5, 639–649. doi: 10.1038/ismej.2010.162, PMID: 20962874 PMC3105739

[ref60] Tan Q. Ma X. Yang B. Liu Y. Xie Y. Wang X.. (2022). Periodontitis pathogen *Porphyromonas gingivalis* promotes pancreatic tumorigenesis via neutrophil elastase from tumor-associated neutrophils. Gut Microbes 14:2073785. doi: 10.1080/19490976.2022.2073785 35549648 PMC9116393

[ref61] Thomas R. M. Gharaibeh R. Z. Gauthier J. Beveridge M. Pope J. L. Guijarro M. V.. (2018). Intestinal microbiota enhances pancreatic carcinogenesis in preclinical models. Carcinogenesis 39, 1068–1078. doi: 10.1093/carcin/bgy073, PMID: 29846515 PMC6067127

[ref62] Thomas F. Hehemann J.-H. Rebuffet E. Czjzek M. Michel G. (2011). Environmental and gut bacteroidetes: the food connection. Front. Microbiol. 2:93. doi: 10.3389/fmicb.2011.00093, PMID: 21747801 PMC3129010

[ref63] Topalian S. L. Hodi F. S. Brahmer J. R. Gettinger S. N. Smith D. C. McDermott D. F.. (2012). Safety, activity, and immune correlates of anti–PD-1 antibody in Cancer. N. Engl. J. Med. 366, 2443–2454. doi: 10.1056/NEJMoa1200690, PMID: 22658127 PMC3544539

[ref64] Turnbaugh P. J. Hamady M. Yatsunenko T. Cantarel B. L. Duncan A. Ley R. E.. (2009). A core gut microbiome in obese and lean twins. Nature 457, 480–484. doi: 10.1038/nature07540, PMID: 19043404 PMC2677729

[ref65] Volant S. Lechat P. Woringer P. Motreff L. Campagne P. Malabat C.. (2020). SHAMAN: a user-friendly website for metataxonomic analysis from raw reads to statistical analysis. BMC bioinformatics 21, 1–15. doi: 10.1186/s12859-020-03666-4 32778056 PMC7430814

[ref66] Walters W. A. Xu Z. Knight R. (2014). Meta-analyses of human gut microbes associated with obesity and IBD. FEBS Lett. 588, 4223–4233. doi: 10.1016/j.febslet.2014.09.039, PMID: 25307765 PMC5050012

[ref67] Wang J. He P. Gaida M. M. Yang S. Schetter A. Gaedcke J.. (2015). Abstract 923: nitric oxide signaling pathway as a pathogenic driver in pancreatic cancer. Cancer Res. 75:923. doi: 10.1158/1538-7445.AM2015-923

[ref68] Wexler H. M. (2007). Bacteroides: the good, the bad, and the nitty-gritty. Clin. Microbiol. Rev. 20, 593–621. doi: 10.1128/CMR.00008-07, PMID: 17934076 PMC2176045

[ref69] Wu G. D. Chen J. Hoffmann C. Bittinger K. Chen Y.-Y. Keilbaugh S. A.. (2011). Linking long-term dietary patterns with gut microbial Enterotypes. Science 334, 105–108. doi: 10.1126/science.1208344, PMID: 21885731 PMC3368382

[ref70] Yatsunenko T. Rey F. E. Manary M. J. Trehan I. Dominguez-Bello M. G. Contreras M.. (2012). Human gut microbiome viewed across age and geography. Nature 486, 222–227. doi: 10.1038/nature11053, PMID: 22699611 PMC3376388

[ref71] Zackular J. P. Baxter N. T. Iverson K. D. Sadler W. D. Petrosino J. F. Chen G. Y.. (2013). The gut microbiome modulates colon tumorigenesis. MBio 4, e00692–e00613. doi: 10.1128/mBio.00692-13, PMID: 24194538 PMC3892781

[ref72] Zambirinis C. P. Pushalkar S. Saxena D. Miller G. (2014). Pancreatic cancer, inflammation, and microbiome. Cancer J. 20, 195–202. doi: 10.1097/PPO.0000000000000045, PMID: 24855007 PMC4112373

[ref73] Zheng Y. Wang T. Tu X. Huang Y. Zhang H. Tan D.. (2019). Gut microbiome affects the response to anti-PD-1 immunotherapy in patients with hepatocellular carcinoma. J. Immunother. Cancer 7:193. doi: 10.1186/s40425-019-0650-9 31337439 PMC6651993

