# Supplementary material for: Metagenomic analysis unveils the microbial landscape of pancreatic tumors
Source: Front Microbiol. 2023 Dec 21;14:1275374. doi: 10.3389/fmicb.2023.1275374 (PMC10764597; doi:10.3389/fmicb.2023.1275374)
Supplement: Supplementary file 1 [file Table_1.DOCX]

**Table S1:** The detail information of patients and associated clinical description along with tumor differentiation stages

| **Tumor Sample** | **Sample ID** | **OUT_table ID** | **Adj. Normal Sample** | **Sample ID** | **OUT_table ID** | **Age** | **Sex** | **Race** | **Dx** | **Tumor size** | **T stage (7th)** | **N+** | **Nodal disease** | **M** | **Stage** | **Neoadjuvant?** | **Regimen** | **treatment effect** | **PN invasion** | **LV invasion** | **Grade** | **Differentiation** |
| --- | --- | --- | --- | --- | --- | --- | --- | --- | --- | --- | --- | --- | --- | --- | --- | --- | --- | --- | --- | --- | --- | --- |
| T 10/31/16 | T1 | T1_S1_L001_R1_001.fastq | N 10/31/16 | T4 | T4_S1_L001_R1_001.fastq | 53 | Female | Black | Ductal adenocarcinoma | 2.5 | 2 | 1 | 1 | X | IIB | 1 | FOLFIRINOX to Gem abraxane | Partial | 1 | 0 | G2 | well to moderate |
| T 10/7/16 | T2 | T2_S1_L001_R1_001.fastq | N 10/7/16 | T5 | T5_S1_L001_R1_001.fastq | 68 | Male | Black | Ductal adenocarcinoma | 4 | 3 | 6 | 1 | X | IIB | 0 | NA | NA | 1 | 1 | G3 | moderate to poor |
| T 10/28/16 | T3 | T3_S1_L001_R1_001.fastq | N 10/28/16 | T6 | T6_S1_L001_R1_001.fastq | 71 | Female | Black | Ductal adenocarcinoma | 1.8 | 1 | 0 | 0 | X | IA | 1 | Gem/abraxane | Partial | 1 | 0 | G2 | Moderate |
| T 9/30/16 | T8 | T8_S1_L001_R1_001.fastq | N 9/30/16 | T7 | T7_S1_L001_R1_001.fastq | 57 | Female | White | Ductal adenocarcinoma | 1.7 | 1 | 0 | 0 | X | IA | 1 | FOLFIRINOX | Prominent | 1 | 0 | G2 | Moderate |
| T 9/8/16 | T10 | T10_S1_L001_R1_001.fastq | N 9/8/16 | T9 | T9_S1_L001_R1_001.fastq | 66 | Female | White | Ductal adenocarcinoma | 4.3 | 3 | 4 | 1 | X | IIB | 0 | NA | NA | 0 | 1 | G3 | Poorly |
| T 9/12/16 | T12 | T12_S1_L001_R1_001.fastq | N 9/12/16 | T11 | T11_S1_L001_R1_001.fastq | 59 | Male | Other | Ductal adenocarcinoma | 2.5 | 3 | 3 | 1 | X | IIB | 1 | FOLFIRINOX | Minimal | 1 | 1 | G1 | Well |
| T 9/1/16 | T16 | T16_S1_L001_R1_001.fastq | N 9/1/16 | T15 | T15_S1_L001_R1_001.fastq | 77 | Female | White | Ductal adenocarcinoma | 6.5 | 3 | 1 | 1 | X | IIB | 0 | NA | NA | 1 | 1 | G2 | Moderate |
| T 2015-003 | T18 | T18_S1_L001_R1_001.fastq | N 2015-003 | T17 | T17_S1_L001_R1_001.fastq | 53 | Male | White | Ductal adenocarcinoma | 4.5 | 3 | 1 | 1 | X | IIB | 0 | NA | NA | 1 | 1 | G3-G4 | Poorly |
| T 2014-009 | T20 | T20_S1_L001_R1_001.fastq | N 2014-009 | T19 | T19_S1_L001_R1_001.fastq | 72 | Male | White | Ductal adenocarcinoma | 3.5 | 2 | 6 | 1 | X | IIB | 0 | NA | NA | 1 | 1 | G2 | Moderate |
| T 11/12/15 | T22 | T22_S1_L001_R1_001.fastq | N 11/12/15 | T21 | T21_S1_L001_R1_001.fastq | 66 | Female | White | Ductal adenocarcinoma | 1.9 | 3 | 4 | 1 | X | IIB | 1 | Gem/abraxane | Partial | 1 | 1 | G1-G2 | Well to moderate |
| T 2015-001 | T24 | T24_S1_L001_R1_001.fastq | N 2015-001 | T23 | T23_S1_L001_R1_001.fastq | 60 | Female | White | Ductal adenocarcinoma | 3 | 3 | 2 | 1 | X | IIB | 0 | NA | NA | 1 | 1 | G2 | Moderate |
| T 3/17/15 | T26 | T26_S1_L001_R1_001.fastq | N 3/17/15 | T25 | T25_S1_L001_R1_001.fastq | 74 | Female | White | Ductal adenocarcinoma | 3 | 3 | 0 | 0 | X | IIA | 0 | NA | NA | 1 | 1 | G2 | Moderate |
| T 10/23/15 | T28 | T28_S1_L001_R1_001.fastq | N 10/23/15 | T27 | T27_S1_L001_R1_001.fastq | 75 | Male | White | Ductal adenocarcinoma | 3 | 3 | 2 | 1 | X | IIB | 0 | NA | NA | 1 | 1 | G1-G2 | Partially well diff. |
| T 5/12/16 | T30 | T30_S1_L001_R1_001.fastq | N 5/12/16 | T29 | T29_S1_L001_R1_001.fastq | 51 | Female | White | Ductal adenocarcinoma | 2.1 | 3 | 1 | 1 | X | IIB | 0 | NA | NA | 1 | 0 | G2 | Moderate |
| T 10/8/15 | T32 | T32_S1_L001_R1_001.fastq | N 10/8/15 | T31 | T31_S1_L001_R1_001.fastq | 78 | Male | White | Ductal adenocarcinoma | 3 | 3 | 3 | 1 | X | IIB | 0 | NA | NA | 1 | 1 | G3 | Poorly |
| T 12/12/16 | T34 | T34_S1_L001_R1_001.fastq | N 12/12/16 | T33 | T33_S1_L001_R1_001.fastq | 57 | Female | White | Ductal adenocarcinoma | 3.5 | 2 | 1 | 1 | X | IIB | 0 | NA | NA | 1 | 0 | G1 | Well |
| T 12/23/16 | T36 | T36_S1_L001_R1_001.fastq | N 12/23/16 | T35 | T35_S1_L001_R1_001.fastq | 67 | Female | Black | Ductal adenocarcinoma | 1.3 | 3 | 0 | 0 | X | IIA | 1 | FOLFIRINOX | Prominent | 1 | 0 | G2 | Moderate |
| T 2015-002 | T38 | T38_S1_L001_R1_001.fastq | N 2015-002 | T37 | T37_S1_L001_R1_001.fastq | 69 | Male | White | Ductal adenocarcinoma | 3 | 3 | 0 | 0 | X | IIA | 0 | NA | NA | 1 | 1 | G2 | Moderate |
| T 8/7/15 | T40 | T40_S1_L001_R1_001.fastq | N 8/7/15 | T39 | T39_S1_L001_R1_001.fastq | 74 | Male | White | Adenosquamous (ductal) | 2 | 1 | 0 | 0 | X | IA | 1 | FOLFIRINOX | | 1 | 0 | G2 | Moderate |
| T 10/09/15 | T14 | T14_S1_L001_R1_001.fastq | N10/09/15 | T13 | T13_S1_L001_R1_001.fastq | 52 | Male | black | Ductal adenocarcinoma | 2.8 | 3 | 3 | 1 |  | IIB | 1 | FOLFIRINOX | Prominent | 1 | 0 | G2 | Moderate |

**N+: Nodal invasion; N1-N3**

**M: Metastasis; M0 means no distant metastasis is present and M1 means evidence of distant metastasis**

**PN invasion: Invasion of nervous structures and nerve sheaths; PN=0 means no invasion, PN=1 means invasion**

**LV invasion: Invasion to lymphatics or blood vessels; LV=0 means no invasion, LV=1 means invasion**
