# Supplementary material for: Metagenomic analysis unveils the microbial landscape of pancreatic tumors
Source: Front Microbiol. 2023 Dec 21;14:1275374. doi: 10.3389/fmicb.2023.1275374 (PMC10764597; doi:10.3389/fmicb.2023.1275374)
Supplement: Supplementary file 3 [file Table_3.DOCX]

>OTU_1

CAAGAGCAGTGCCAGCAGCGGCGGTAAGACAGAGGATGCAAGCGTTATCCGGAATGATTGGGCGTAAAGC

GTCTGTAGGTGGCTTTTCAAGTCCGCCGTCAAATCCCCGGGCTCAACCCTGGACAGGCAGTGGAAACTAC

CAAGCTGGAGTACGGTAGGGGCAGAGGGAATTTCCGGTGGAGCGGTGAAATGCGTTGAGATCGGAAAGAA

CACCAACGGCGAAAGCACTCTGCTGGGCCGACACTGACACTGAGAGACGAAAGCTAGGGGAGCAAATGGG

ATTAGATACCCCAGTAGTCCT

>OTU_2

CAAGAGCAGTGCCAGCAGCGGCGGTAATACGTAGGTCCCGAGCGTTGTCCGGATTTATTGGGCGTAAAGC

GAGCGCAGGCGGTTAGAAAAGTCTGAAGTGAAAGGCAGTGGCTCAACCATTGTAGGCTTTGGAAACTGTT

TAACTTGAGTGCAGAAGGGGAGAGTGGAATTCCATGTGTAGCGGTGAAATGCGTAGATATATGGAGGAAC

ACCGGTGGCGAAAGCGGCTCTCTGGTCTGTAACTGACGCTGAGGCTCGAAAGCGTGGGGAGCGAACAGGA

TTAGATACCCTGGTAGTCCAC

>OTU_3

CACAGTGTGTGCCAGCAGCAGCGGTAATACATAGGGGGCGAGCGTTATCCGGATTTACTGGGCGTAAAGG

GTGCGTAGGTGGTTATAAAAGTTTGTGGTGTAAGTGCAGTGCTTAACGCTGTGAGGCTATGAAAACTATA

TAACTAGAGTGAGACAGAGGCAAGTGGAATTCCATGTGTAGCGGTAAAATGCGTAAATATATGGAGGAAC

ACCAGTGGCGAAGGCGGCTTGCTGGGTCTATACTGACACTGATGCACGAAAGCGTGGGGAGCAAACAGGA

TTAGATACCCTGGTAGTCCAC

>OTU_4

CAAGAGCAGTGCCAGCAGCGGCGGTAATACGGAAGGTCCTGGCGTTATCCGGATTTATTGGGTTTAAAGG

GAGCGCAGGCCGCCCCTTAAGCGTGTTGTGAAACCCGGGCGCCCAACGCCCGGCCTGCAGCGCGAACTGG

GGGGCTTGAGTGCGCGCAACGCCGGCGGAATTCGTCGTGTAGCGGTGAAATGCTTAGATATGACGAGGAA

CCCCGATTGCGAAGGCAGCCGGCGGGAGCGCAACTGACGCTTATGCTCGAAGGCGCGGGTATCGAACAGG

ATTAGATACCCTGGTAGTCCG

>OTU_5

CAACGATCGTGCCAGCAGCGGCGGTAATACAGAGGGTGCGAGCGTTAATCGGATTTACTGGGCGTAAAGC

GTGCGTAGGCGGCTTTTTAAGTCGGATGTGAAATCCCCGAGCTTAACTTGGGAATTGCATTCGATACTGG

GAAGCTAGAGTATGGGAGAGGATGGTAGAATTCCAGGTGTAGCGGTGAAATGCGTAGAGATCTGGAGGAA

TACCGATGGCGAAGGCAGCCATCTGGCCTAATACTGACGCTGAGGTACGAAAGCATGGGGAGCAAACAGG

ATTAGATACCCTGGTAGTCCG

>OTU_6

CACAGACTGTGCCAGCAGCCGCGGTAATACGGAGGGTGCAAGCGTTATCCGGATTTATTGGGTTTAAAGG

GTCCGTAGGCGGACTTATAAGTCAGTGGTGAAAGCCTGTCGCTTAACGATAGAACTGCCATTGATACTGT

AAGTCTTGAGTATATTTGAGGTAGCTGGAATAAGTAGTGTAGCGGTGAAATGCATAGATATTACTTAGAA

CACCAATTGCGAAGGCAGGTTACCAAGATATAACTGACGCTGAGGGACGAAAGCGTGGGGAGCGAACAGG

ATTAGATACCCTGGTAGTCCG

>OTU_7

CACAGACTGTGCCAGCAGCTGCGGTAATACGTAGGGTGCGAGCGTTGTCCGGAATTACTGGGCGTAAAGA

GCTCGTAGGTGGTTTGTCGCGTCGTCTGTGAAATTCCGGGGCTTAACTTCGGGCGTGCAGGCGATACGGG

CATAACTTGAGTGCTGTAGGGGAGACTGGAATTCCTGGTGTAGCGGTGGAATGCGCAGATATCAGGAGGA

ACACCGATGGCGAAGGCAGGTCTCTGGGCAGTAACTGACGCTGAGGAGCGAAAGCATGGGGAGCGAACAG

GATTAGATACCCTGGTAGTCC

>OTU_8

CAACTGCAGTGCCAGCAGCAGCGGTGATACGTAGGGTGCGAGCGTTGTCCGGATTTATTGGGCGTAAAGG

GCTCGTAGGTGGTTGATCGCGTCGGAAGTGTAATCTTGGGGCTTAACCCTGAGCGTGCTTTCGATACGGG

TTGACTTGAGGAAGGTAGGGGAGAATGGAATTCCTGGTGGAGCGGTGGAATGCGCAGATATCAGGAGGAA

CACCAGTGGCGAAGGCGGTTCTCTGGGCCTTTCCTGACGCTGAGGAGCGAAAGCGTGGGGAGCGAACAGG

CTTAGATACCCTGGTAGTCCC

>OTU_9

CAAGCATCGTGCCAGCCGCGGCGGTAATACGTAGGGTGCGAGCGTTAATCGGAATTACTGGGCGTAAAGC

GTGCGCAGGCGGTTATGTAAGACAGATGTGAAATCCCCGGGCTCAACCTGGGAACTGCATTTGTGACTGC

ATGGCTAGAGTACGGTAGAGGGGGATGGAATTCCGCGTGTAGCAGTGAAATGCGTAGATATGCGGAGGAA

CACCGATGGCGAAGGCAATCCCCTGGACCTGTACTGACGCTCATGCACGAAAGCGTGGGGAGCAAACAGG

ATTAGATACCCTGGTAGTCCG

>OTU_10

CACAGACTGTGCCAGCAGCTGCGGTAATACGTAGGGTGCAAGCGTTAATCGGAATTACTGGGCGTAAAGC

GTGCGCAGGCGGTTTTGTAAGTCTGACGTGAAATCCCCGGGCTCAACCTGGGAATTGCGTTGGAGACTGC

AAGGCTAGAATCTGGCAGAGGGGGGTAGAATTCCACGTGTAGCAGTGAAATGCGTAGAGATGTGGAGGAA

CACCGATGGCGAAGGCAGCCCCCTGGGTCAAGATTGACGCTCATGCACGAAAGCGTGGGGAGCAAACAGG

ATTAGATACCCTGGTAGTCCC

>OTU_11

CACAACTGGTGCCAGCAGCAGCGGTAATACGAAGGGTGCAAGCGTTACTCGGAATTACTGGGCGTAAAGC

GTGCGTAGGTGGTTATTTAAGTCCGTTGTGAAAGCCCTGGGCTCAACCTGGGAACTGCAGTGGATACTGG

ATGACTAGAATGTGGTAGAGGGTAGCGGAATTCCTGGTGTAGCAGTGAAATGCGTAGAGATCAGGAGGAA

CATCCATGGCGAAGGCAGCTACCTGGACCAACATTGACACTGAGGCACGAAAGCGTGGGGAGCAAACAGG

ATTAGATACCCTGGTAGTCCC

>OTU_12

CACAGTGTGTGCCAGCAGCTGCGGTAATACATAGGTGGCAAGCGTTATCCGGATTTATTGGGCGTATAGG

GTGCGTAGGCGGTTTTGCAAGTTTGAGGTTAAAGCCCGGAGCTCAACTCCGGTCCGCCTTGAAAACTGCA

TTACTAGAATGCAAGAGAGGTAAGTGGAATTCCATGTGTAGCGGTGAAATGCGTAGATATATGGAAGAAC

ACCTGTGGCGAAAGCGGCTTACTGGCTTGTTATTGACGCTGAGGCACGAAAGCGTGGGGAGCAAATAGGA

TTAGATACCCTAGTAGTCCAC

>OTU_13

CAAGCATCGTGCCAGCAGCTGCGGTAATACGTAGGGTGCAAGCGTTAATCGGAATTACTGGGCGTAAAGC

GTGCGCAGGCGGTTTTGTAAGTCTGATGTGAAATCCCCGGGCTCAACCTGGGAACTGCATTGGAGACTGC

AAGGCTAGAGTGTGTCAGAGGGGGGTAGAATTCCACGTGTAGCAGTGAAATGCGTAGAGATGTGGAGGAA

TACCGATGGCGAAGGCAGCCCCCTGGGATAACACTGACGCTCATGCACGAAAGCGTGGGGAGCAAACAGG

ATTAGATACCCTGGTAGTCCC

>OTU_14

CACAGACTGTGCCAGCAGCTGCGGTAATACAGAGGGTGCGAGCGTTAATCGGAATTACTGGGCGTAAAGC

GAGTGTAGGTGGCTCATTAAGTCACATGTGAAATCCCCGGGCTTAACCTGGGAACTGCATGTGATACTGG

TGGTGCTAGAATATGTGAGAGGGAAGTAGAATTCCAGGTGTAGCGGTGAAATGCGTAGAGATCTGGAGGA

ATACCGATGGCGAAGGCAGCTTCCTGGCATAATATTGACACTGAGATTCGAAAGCGTGGGTAGCAAACAG

GATTAGATACCCTGGTAGTCC

>OTU_15

CACAACTGGTGCCAGCAGCGGCGGTAAGACGGAGGTGGCAAGCGTTGCTCGGATTCACTGGGCGTAAAGG

GCACGTAGGCGGTCCCGTACGCCCCACCTGAAAGGCTCTGGCTTAACCAGAGCAGGCGGTGGGGGACTGC

GGGACTGGAGGGTGGGAGAGGCGAGTGGAATTCCCGGTGTAAGGGTGAAATCTGTAGAGATCGGGAGGAA

CACCAGTGGCGAAAGCGGCTCGCTGGCCCATACCTGACGCTGAGGTGCGAAAGCCAGGGGAGCAAACAGG

ATTAGATACCCTGGTAGTCCT

>OTU_16

CAAGACCTGTGCCAGCCGCTGCGGTAATACAGAGGATGCAAGCGTTATCCGGAATGATTGGGCGTAAAGC

GTCTGTAGGTGGCTTTTTAAGTCCGCCGTCAAATCCCAGGGCTCAACCCTGGACAGGCGGTGGAAACTAC

CAAGCTGGAGTACGGTAGGGGCAGAGGGAATTTCCGGTGGAGCGGTGAAATGCGTAGAGATCGGAAAGAA

CACCAACGGCGAAAGCACTCTGCTGGGCCGACACTGACACTGAGAGACGAAAGCTAGGGGAGCGAATGGG

ATTAGATACCCCAGTAGTCCT

>OTU_17

CAAGAGCAGTGCCAGCAGCGGCGGTAATACggggggggCAAGTGTTATTCGGAATGACTGGGCGTAAAGG

GCACGTAGGCGGTGAAAAGGGTGGAAAGTGAAAGTCGCCAAAACACTGGCGGGGTGCTTTCTTGACCATT

TCACTTGAGTGAGATAGGGGAGAGTGGAATTTCGTGTGGAGGGATCAAATCCTAAGATATACGAAGGAAC

GCCAACCGCGAAGGCAGCTCTCTGGGTCCCCACTGACGCTGGGGTGCGAAAGCATGGGGAGCAAACAGGA

TCAGATACCCTGGTAGTCCAT

>OTU_18

CAACTGCAGTGCCAGCAGCTGCGGTAATACGGAGGATGCAAGCGTTATCCGGAATTATTGGGCGTAAAGC

GTCCGCAGGTGGCAATTCAAGTCTGCTGTCAAAGGTTCTGGCTCAACCAGAAACAGGCAGTGGAAACTGA

ATAGCTAGAGTGCGGTAGGGGCAGAGGGAATTCCCAGTGTAGCGGTGAAATGCGTAGAGATTGGGAAGAA

CACCGGTGGCGAAAGCGCTCTGCTAGGCCGTAACTGACACTCATGGACGAAAGCTAGGGGAGCGAATGGG

ATTAGATACCCCAGTAGTCCT

>OTU_19

CAAGACGAGTGCCAGCAGCGGCGGTAAGACggggggggCAAGTGTTATTCGGAATGACTAGGCGTAAAGG

GCACGTAGGCGGTGAATCGGGTTGGAAGTGAAAGTCGCCAACAATTGGCGGGGTGCTTTCGGAACCAATT

CACTTGAGTGAGATAGGGGAGAGTGGAATTTCGTGTGTAGGGATCAAATCCGGAGATCTACGAAGGAACG

CCAAAAGCGAAGGCAGCTCTCTGGGTCTACACTGACGCTGGGGTGCGAAAGCATGGGGAGCAAACAGGAT

CAGATACCCTGGTAGTCCATG

>OTU_20

CAAGACGAGTGCCAGCCGCTGCGGTAAAACAGAGGATGCAAGCGTTATCCGGAATTATTGGGCGTAAAGT

GTCTGTAGGTGGCTTTTCAAGTCCGTCGTCAAATCCCAGGGCTCAACCCTGGACAGGCGGTGGAAACTAC

CAAGCTAGAGTACGGTAGAGGCAGAGGGAATTTCCGGTGGAGCGGTGAAATGCGTTGAGATCGGGAAGAA

CACCAACAGGCGAAAGCACTCTGCTGGGCCGTCACTGACACTCAGAGACGAAAGCTAGGGGAGCGAATGG

GATTAGATACCCCAGTAGTCC

>OTU_21

CAACGTACGTGCCAGCAGCGGCGGTAATACGTAGGTCCCGAGCGTTGTCCGGATTTATTGGGCGTAAAGC

GAGCGCAGGCGGTTAGATAAGTCTGAAGTTAAAGGCTGTGGCTTAACCATAGTACGCTTTGGAAACTGTT

TAACTTGAGTGCAAGAGGGGAGAGTGGAATTCCATGTGTAGCGGTGAAATGCGTAGATATATGGAGGAAC

ACCGGTGGCGAAAGCGGCTCTCTGGCTTGTAACTGACGCTGAGGCTCGAAAGCGTGGGGAGCAAACAGGA

TTAGATACCCTGGTAGTCCAC

>OTU_22

CAAGAGCAGTGCCAGCAGCGGCGGTAATACGTAGGTGGCAAGCGTTGTCCGGAATTATTGGGCGTAAAGC

GCGCGCAGGCGGATCAGTCAGTCTGTCTTAAAAGTTCGGGGCTTAACCCCGTGATGGGATGGAAACTGCT

GATCTAGAGTATCGGAGAGGAAAGTGGAATTCCTAGTGTAGCGGTGAAATGCGTAGATATTAGGAAGAAC

ACCAGTGGCGAAGGCGACTTTCTGGACGAAAACTGACGCTGAGGCGCGAAAGCCAGGGGAGCGAACGGGA

TTAGATACCCCGGTAGTCCTG

>OTU_23

CAAGAGCAGTGCCAGCCGCAGCGGTAATACGTAGGGTGCGAGCGTTGTCCGGAATTACTGGGCGTAAAGA

GCTCGTAGGTGGTTTGTCGCGTCGTCTGTGAAATTCCGGGGCTTAACTCCGGGCGTGCAGGCGATACGGG

CATAACTTGAGTGCTGTAGGGGAGACTGGAATTCCTGGTGTAGCGGTGAAATGCGCAGATATCAGGAGGA

ACACCGATGGCGAAGGCAGGTCTCTGGGCAGTAACTGACGCTGAGGAGCGAAAGCATGGGTAGCGAACAG

GATTAGATACCCTGGTAGTCC

>OTU_24

CAAGAGCAGTGCCAGCAGCGGCGGTAATACGTATGTCACGAGCGTTATCCGGATTTATTGGGCGTAAAGC

GCGTCTAGGTGGTTATGTAAGTCTGATGTGAAAATGCAGGGCTCAACTCTGTATTGCGTTGGAAACTGTA

TAACTAGAGTACTGGAGAGGTAAGCGGAACTACAAGTGTAGAGGTGAAATTCGTAGATATTTGTAGGAAT

GCCGATGGGGAAGCCAGCTTACTGGACAGATACTGACGCTAAAGCGCGAAAGCGTGGGTAGCAAACAGGA

TTAGATACCCTGGTAGTCCAC

>OTU_25

CAAGTGGAGTGCCAGCAGCCGCGGTAAGACGTAGGATGCGAGCGTTGTCCGGATTTATTGGGCGTAAAGA

GTTCGTAGGTGGTTTGTTAAGTTTGGTGTTAAAGATCGGGGCTCAACCCTGGGACTGCACTGAATACTGG

CAGACTCGAGTGTGGTAGAGGCTAGTGGAATTCCCAGTGTAGCGGTGAAATGCGTAGATATTGGGAAGAA

CACCGGTGGCGTAGGCGACTAGCTGGGCCATAACTGACGCTGAGGAACGAAAGCCAGGGGAGCGAATGGG

ATTAGATACCCCAGTAGTCCT

>OTU_26

CAAGACGAGTGCCAGCAGCTGCGGTAATACGAAGGGGGCTAGCGTTGCTCGGAATCACTGGGCGTAAAGG

GCGCGTAGGCGGCCGATTAAGTCGGGGGTGAAAGCCTGTGGCTCAACCACAGAATTGCCTTCGATACTGG

TTGGCTTGAGACCGGAAGAGGACAGCGGAACTGCGAGTGTAGAGGTGAAATTCGTAGATATTCGCAAGAA

CACCAGTGGCGAAGGCGGCTGTCTGGTCCGGTTCTGACGCTGAGGCGCGAAAGCGTGGGGAGCAAACAGG

ATTAGATACCCTGGTAGTCCC

>OTU_27

CAAGTGGAGTGCCAGCAGCAGCGGTAATACGTATGTCGCAAGCGTTATCCGGATTTATTGGGCGTAAAGC

GCGTCTAGGCGGAAAAATAAGTCTGATGTTAAAATGCGGGGCTCAACTCCGTATTGCGTTGGAAACTGTT

TTTCTAGAGTACTGGAGAGGTGGGCGGAACTACAAGTGTAGAGGTGAAATTCGTAGATATTTGTAGGAAT

GCCGATGGAGAAGTCAGCTCACTGGACAGATACTGACGCTAAAGCGCGAAAGCGTGGGGAGCAAACAGGA

TTAGATACCCTGGTAGTCCAC

>OTU_28

CAACTGGTGTGCCAGCAGCGGCGGTAATACGTAGGTCCCGAGCGTTGTCCGGATTTATTGGGCGTAAAGC

GAGCGCAGGCGGTTTAATAAGTCTGAAGTTAAAGGCAGTGGCTTAACCATTGTTCGCTTTGGAAACTGTT

AAACTTGAGTGCAGAAGGGGAGAGTGGAATTCCATGTGTAGCGGTGAAATGCGTAGATATATGGAGGAAC

ACCGGTGGCGAAAGCGGCTCTCTGGTCTGTAACTGACGCTGAGGCTCGAAAGCGTGGGGAGCAAACAGGA

TTAGATACCCTGGTAGTCCAC

>OTU_29

CAACTGCAGTGCCAGCAGCGGCGGTAATACGTAGGTGACAAGCGTTGTCCGGAATTATTGGGCGTAAAGG

GAGCGCAGGCGGTTGGAATAGTCTGATGTGAAAGCCCACGGCTTAACCGTGGAATTGCATTGGAAACTGT

CCAACTTGAATGCAGAAGAGGAAAGCGGAATTCCATGTGTAGCGGTGAAATGCGTAGATATATGGAGGAA

CACCAGTGGCGAAGGCGGCTTTCTGGTCTGTGATTGACGCTGAGGCTCGAAAGCGTGGGGAGCGAACAGG

ATTAGATACCCTGGTAGTCCC

>OTU_30

CAAGACGAGTGCCAGCAGCTGCGGTAATACGTAGGGGCCAAGCGTTGTCCGGATTTATTGGGCGTAAAGA

GCTCGTAGGCGGTTCGGTAAGTCAGCTGTGAAATCTCCACGCTCAACGTGGAGGGGTCAGCTGATACTGC

CGTGACTTGAGTCTGGTAGGGGAGTGCGGAATTCCCGGTGTAGCGGTGAAATGCGCAGATATCGGGAGGA

ACACCAGTGGCGAAGGCGGCACTCTGGGCCAGTACTGACGCTGAGGAGCGAAAGCGTGGGGAGCGAACAG

GATTAGATACCCTGGTAGTCC

>OTU_31

CACAGTGTGCCAGCCGCAGCGGTAATACGTAGGTGGCAAGCGTTATCCGGAATTATTGGGCGTAAAGCGC

GCGTAGGCGGTTTTTTAAGTCTGATGTGAAAGCCCACGGCTCAACCGTGGAGGGTCATTGGAAACTGGAA

AACTTGAGTGCAGAAGAGGAAAGTGGAATTCCATGTGTAGCGGTGAAATGCGCAGAGATATGGAGGAACA

CCAGTGGCGAAGGCGACTTTCTGGTCTGTAACTGACGCTGATGTGCGAAAGCGTGGGGATCAAACAGGAT

TAGATACCCTGGTAGTCCACG

>OTU_32

CACAGTGTGTGCCAGCAGCAGCGGTAATACATAGGTTGCAAGCGTTATCCGGAATTATTGGGCGTAAAGC

GTCTGTAGGTTGTTTGTTAAGTCTGGCGTTAAATTTTGGGGCTCAACCCCAAAACGCGTTGGATACTGGC

AGGCTAGAGTTGTGTAGAGGTTAGCGGAATTCCTTGTGAAGCGGTGAAATGCGTAGATATAAGGAAGAAC

ACCAAGATGGCGAAGGCAGCTAACTGGACATATACTGACACTGAGAGACGAAAGCGTGGGGAGCAAACAG

GATTAGATACCCTGGTAGTCC

>OTU_33

CAAGCAAGGTGCCAGCAGCTGCGGTAATACGTAGGGTGCAAGCGTTGTCCGGAATTACTGGGCGTAAAGA

GCTCGTAGGTGGTTTGTCGCGTCGTTTGTGGAATACCGCAGCTTAACTGTGGGGTTGCAGGCGATACGGG

CATAACTTGAGTGCTGTAGGGGAGACTGGAATTCCTGGTGTAGCGGTGGAATGCGCAGATATCAGGAGGA

ACACCGATGGCGAAGGCAGGTCTCTGGGCAGTAACTGACGCTGAGGAGCGAAAGCATGGGTAGCGAACAG

GATTAGATACCCTGGTAGTCC

>OTU_34

CACAACTGGTGCCAGCAGCAGCGGTAATACGGAGGTGGCAAGCGTTGCTCGGATTTACTGGGTGTAAAGG

GCAAGTAGGCGGCTTCGTAAGTCGGGTGTGAAATCCCACGGCTTAACCGTGGAACTGCGCCCGAAACTGC

GGAGCTTGAGGACAGGAGAGGCGAAGGGAATTCCCGGTGTAAGGGTGAAATCTGTAGAGATCGGGAGGAA

CACCAGTGGCGAAGGCGCTTCGCTGGCCTGTCCCTGACGCTGAGAGGCGAAAGCTAGGGGAGCAAACAGG

ATTAGATACCCTGGTAGTCCT

>OTU_35

CAACGAAGGTGCCAGCAGCGGCGGTAATACGTAGGGTGCGAGCGTTGTCCGGAATTACTGGGCGTAAAGA

GCTCGTAGGTGGTCTGTCGCGTCATTTGTGAAAGCCCGGGGCTTAACTCCGGGTTGGCAGGTGATACGGG

CATGACTGGAGTACTGTAGGGGAGACTGGAATTCCTGGTGTAGCGGTGAAATGCGCAGATATCAGGAGGA

ACACCGGTGGCGAAGGCGGGTCTCTGGGCAGTAACTGACGCTGAGGAGCGAAAGCATGGGTAGCGAACAG

GATTAGATACCCTGGTAGTCC

>OTU_36

CAACGTTGGTGCCAGCAGCGGCGGTAATACGTAGGTGGCGAGCGTTGTCCGGAATTATTGGGCGTAAAGA

GCATGTAGGCGGCTTAATAAGTCGAGCGTGAAAATGCGGGGCTCAACCCCGTATGGCGCTGGAAACTGTT

AGGCTTGAGTGCAGGAGAGGAAAGGGGAATTCCCAGTGTAGCGGTGAAATGCGTAGATATTGGGAGGAAC

ACCAGTGGCGAAGGCGCCTTTCTGGACTGTGTCTGACGCTGAGATGCGAAAGCCAGGGTAGCGAACGGGA

TTAGATACCCCGGTAGTCCTG

>OTU_37

CAACTCGAGTGCCAGCCGCCGCGGTAATACGTAGGGTGCAAGCGTTAATCGGAATTACTGGGCGTAAAGC

GTGCGCAGGCGGTTTTGTAAGTCTGACGTGAAAGCCCCGGGCTCAACCTGGGAATTGCGTTGGAGACTGC

AAGGCTTGAATCTGGCAGAGGGGGGTAGAATTCCACGTGTAGCAGTGAAATGCGTAGAGATGTGGAGGAA

CACCGATGGCGAAGGCAGCCCCCTGGGTCAAGATTGACGCTCATGCACGAAAGCGTGGGGAGCAAACAGG

ATTAGATACCCTGGTAGTCCG

>OTU_38

CAAGCAAGGTGCCAGCAGCGGCGGTAATACGGAGGGTGCAAGCGTTAATCGGAATTACTGGGCGTAAAGC

GCACGCAGGCGGTCTGTCAAGTCGGATGTGAAATCCCCGGGCTCAACCTGGGAACTGCATTCGAAACTGG

CAGGCTAGAGTCTTGTAGAGGGGGGTAGAATTCCAGGTGTAGCGGTGAAATGCGTAGAGATCTGGAGGAA

TACCGGTGGCGAAGGCGGCCCCCTGGACAAAGACTGACGCTCAGGTGCGAAAGCGTGGGGAGCAAACAGG

ATTAGATACCCTGGTAGTCC

>OTU_39

CAACTGCAGTGCCAGCAGCGGCGGTAATACGAAGGGTGCAAGCGTTAATCGGAATTACTGGGCGTAAAGC

GCGCGTAGGTGGTTCGTTAAGTTGGATGTGAAAGCCCCGGGCTCAACCTGGGAACTGCATCCAAAACTGG

CGAGCTAGAGTACGGTAGAGGGTGGTGGAATTTCCTGTGTAGCGGTGAAATGCGTAGATATAGGAAGGAA

CACCAGTGGCGAAGGCGACCACCTGGACTGATACTGACACTGAGGTGCGAAAGCGTGGGGAGCAAACAGG

ATTAGATACCCTGGTAGTCC

>OTU_40

CAAGCATCGTGCCAGCAGCGGCGGTAATACGTAGGTGGCAAGCGTTATCCGGAATTATTGGGCGTAAAGC

GCGCGTAGGCGGTTTCTTAAGTCTGATGTGAAAGCCCACGGCTCAACCGTGGAGGGTCATTGGAAACTGG

GAGACTTGAGTGCAGAAGAGGAAAGTGGAATTCCATGTGTAGCGGTGAAATGCGCAGAGATATGGAGGAA

CACCAGTGGCGAAGGCGACTTTCTGGTCTGTAACTGACGCTGATGTGCGAAAGCGTGGGGATCAAACAGG

ATTAGATACCCTGGTAGTCC

>OTU_41

CAACGTACGTGCCAGCAGCGGCGGTAATACGGAGGGAGCTAGCGTTGTTCGGAATTACTGGGCGTAAAGC

GCACGTAGGCGGCGACACAAGTCAGAGGTGAAAGCCCGGGGCTCAACCCCGGAACTGCCTTTGAAACTAG

GTTGCTAGAATCTTGGAGAGGTCAGTGGAATTCCGAGTGTAGAGGTGAAATTCGTAGATATTCGGAAGAA

CACCAGTGGCGAAGGCGACTGACTGGACAAGTATTGACGCTGAGGTGCGAAAGCGTGGGGAGCAAACAGG

ATTAGATACCCTGGTAGTCC

>OTU_42

CACAGTGTGTGCCAGCAGCTGCGGTAATACATAGGTCGCAAGCGTTATCCGGAATTATTGGGCGTAAAGC

GTCCGTAGGTTTTTTGCTAAGTCTGGAGTTAAATGCTGAAGCTCAACTTCAGTCCGCTTTGGATACTGGC

AAAATAGAATTATAAAGAGGTTAGCGGAATTCCTAGTGAAGCGGTGGAATGCGTAGATATTAGGAAGAAC

ACCAATAGGCGAAGGCAGCTAACTGGTTATATATTGACACTAAGGGACGAAAGCGTGGGGAGCAAACAGG

ATTAGATACCCTGGTAGTCC

>OTU_43

CAAGTCGTGTGCCAGCAGCGGCGGTAATACAGAGGGTGCAAGCGTTAATCGGAATTACTGGGCGTAAAGC

GCGCGTAGGTGGTTCGTTAAGTTGGATGTGAAATCCCCGGGCTCAACCTGGGAACTGCATTCAAAACTGA

CGAGCTAGAGTATGGTAGAGGGTGGTGGAATTTCCTGTGTAGCGGTGAAATGCGTAGATATAGGAAGGAA

CACCAGTGGCGAAGGCGACCACCTGGACTGATACTGACACTGAGGTGCGAAAGCGTGGGGAGCAAACAGG

ATTAGATACCCTGGTAGTCC

>OTU_44

CACAGACTGTGCCAGCAGCTGCGGTAATACGGAGGGTGCAAGCGTTATCCGGATTTATTGGGTTTAAAGG

GTCCGTAGGCGGATTAGTAAGTCAGTGGTGAAAGCCCGCAGCTCAACTGTGGAACTGCCATTGATACTGC

TAGTCTTGAGTGTAGTTGAAGTAGCTGGAATGAGTAGTGTAGCGGTGAAATGCATAGATATTACTCAGAA

CACCAATTGCGAAGGCAGGTTACTAAGTTACAACTGACGCTGATGGACGAAAGCGTGGGGAGCGAACAGG

ATTAGATACCCTGGTAGTCA

>OTU_45

CACAACTGGTGCCAGCAGCGGCGGTAATACGTAGGGTGCAAGCGTTAATCGGAATTACTGGGCGTAAAGC

GTGCGCAGGCGGTTATGCAAGACAGAGGTGAAATCCCCGGGCTCAACCTGGGAACTGCCTTTGTGACTGC

ATGGCTAGAGTACGGTAGAGGGGGATGGAATTCCGCGTGTAGCAGTGAAATGCGTAGATATGCGGAGGAA

CACCGATGGCGAAGGCAATCCCCTGGACCTGTACTGACGCTCATGCACGAAAGCGTGGGGAGCAAACAGG

ATTAGATACCCTGGTAGTCC

>OTU_46

CAACTGCAGTGCCAGCAGCGGCGGTAATACGGAGGGCGCGAGCGTTACCCGGATTTACTGGGCGTAAAGG

GCGTGTAGGCGGCCTGGGGCGTCCCATGTGAAAGGCCACGGCTCAACCGTGGAGGAGCGTGGGATACGCT

CAGGCTAGAGGGTGGGAGAGGGTGGTGGAATTCCCGGAGTAGCGGTGAAATGCGCAGATACCGGGAGGAA

CGCCGATGGCGAAGGCAGCCACCTGGTCCACTTCTGACGCTGAGGCGCGAAAGCGTGGGGAGCAAACCGG

ATTAGATACCCGGGTAGTCC

>OTU_47

CAACTGCAGTGCCAGCAGCCGCGGTAATACGTAGGTGGCAAGCGTTATCCGGAATTATTGGGCGTAAAGC

GCGCGTAGGCGGTTTTTTAAGTCTGATGTGAAAGCCCACGGCTCAACCATGGAGGGTCATTGGAAACTGG

AAAACTTGAGTGCAGAAGAGGAAAGTGGAATTCCATGTGTAGCGGTGAAATGCGCAGAGATATGGAGGAA

CACCAGTGGCGAAGGCGACTTTCTGGTCTGTAACTGACGCTGATGTGCGAAAGCGTGGGGATCAAACAGG

ATTAGATACCCTGGTAGTCC

>OTU_48

CAACACGTGTGCCAGCAGCGGCGGTAATACGTAGGGTGCAAGCGTTGTCCGGAATTATTGGGCGTAAAGA

GCTCGTAGGCGGTTTGTCGCGTCTGCTGTGAAAACCCGAGGCTCAACCTCGGGCCTGCAGTGGGTACGGG

CAGACTAGAGTGCGGTAGGGGAGATTGGAACTCCTGGTGTAGCGGTGGAATGCGCAGATATCAGGAAGAA

CACCGATGGCGAAGGCAGATCTCTGGGCCGTTACTGACGCTGAGGAGCGAAAGCATGGGGAGCGAACAGG

ATTAGATACCCTGGTAGTCC

>OTU_49

CAAGAGCAGTGCCAGCAGCGGCGGTAATACGAAAGGTGCGAGCGTTAATCGGAATTACTGGGCGTAAAGC

GCGCGTAGGTGGTGTGTTAAGTCGGATGTGAAAGCCCTGGGCTCAACCTGGGAATGGCATCCGATACTGG

CCCGCTAGAGTGCAGTAGAGGGAGGTGGAATTTCCGGTGTAGCGGTGAAATGCGTAGAGATCGGAAGGAA

CACCAGTGGCGAAGGCGGCCTCCTGGACTGACACTGACACTGAGGTGCGAAAGCGTGGGGAGCGAACAGG

ATTAGATACCCTGGTAGTCC

>OTU_50

CACAACACGTGCCAGCAGCTGCGGTAATACGTAGGGTGCAAGCGTTAATCGGAATTACTGGGCGTAAAGC

GTGCGCAGGCGGTTCCATAAGACAGATGTGAAATCCCCGGGCTCAACCTGGGAACTGCATTTGTGACTGT

GGAGCTAGAGTACGGTAGAGGGGGATGGAATTCCGCGTGTAGCAGTGAAATGCGTAGATATGCGGAGGAA

CACCAATGGCGAAGGCAATCCCCTGGACCTGTACTGACGCTCATGCACGAAAGCGTGGGGAGCAAACAGG

ATTAGATACCCTGGTAGTCC

>OTU_51

CAACCTTCGTGCCAGCAGCTGCGGTAATACGGAGGGTGCAAGCGTTAATCGGAATTACTGGGCGTAAAGC

GCACGCAGGCGGTTTGTTAAGTCAGATGTGAAATCCCCGGGCTCAACCTGGGAACTGCATTTGAAACTGG

CAAGCTTGAGTCTTGTAGAGGGGGGTAGAATTCCAGGTGTAGCGGTGAAATGCGTAGAGATCTGGAGGAA

TACCGGTGGCGAAGGCGGCCCCCTGGACAAAGACTGACGCTCAGGTGCGAAAGCGTGGGGAGCAAACAGG

ATTAGATACCCTGGTAGTCC

>OTU_52

CACAGACTGTGCCAGCAGCAGCGGTAATACGGAGGATCCGAGCGTTATCCGGATTTATTGGGTTTAAAGG

GTGCGCAGGCGGCATGTTAAGTCGGCGGTGAAATTTTGCAGCTCAACTGTAAAAGAGCCTTCGAAACTGG

CAAGCTTGAGTGTGGATGAAGTAGGCGGAATTTGTGGTGTAGCGGTGAAATGCATAGATATCACAAAGAA

CACCGATTGCGCAGGCAGCTTACTAAACCATAACTGACGCTCATGCACGAAGGCGTGGGGATCAAACAGG

ATTAGATACCCTGGTAGTCC

>OTU_53

CACAACTGGTGCCAGCAGCGGCGGTAATACGAAGGGGGCTAGCGTTGCTCGGAATCACTGGGCGTAAAGG

GCGCGTAGGCGGCGTTTTAAGTCGGGGGTGAAAGCCTGTGGCTCAACCACAGAATGGCCTTCGATACTGG

GACGCTTGAGTATGGTAGAGGTTGGTGGAACTGCGAGTGTAGAGGTGAAATTCGTAGATATTCGCAAGAA

CACCGGTGGCGAAGGCGGCCAACTGGACCATTACTGACGCTGAGGCGCGAAAGCGTGGGGAGCAAACAGG

ATTAGATACCCTGGTAGTCC

>OTU_54

CAACTCGAGTGCCAGCAGCGGCGGTAATACGTAGGGTGCAAGCGTTAATCGGAATTACTGGGCGTAAAGC

GTGCGCAGGCGGTTCGGAAAGAAAGATGTGAAATCCCAGAGCTTAACTTTGGAACTGCATTTTTAACTAC

CGAGCTAGAGTGTGTCAGAGGGAGGTGGAATTCCGCGTGTAGCAGTGAAATGCGTAGATATGCGGAGGAA

CACCGATGGCGAAGGCAGCCTCCTGGGATAACACTGACGCTCATGCACGAAAGCGTGGGGAGCAAACAGG

ATTAGATACCCTGGTAGTCC

>OTU_55

CAACGTACGTGCCAGCAGCGGCGGTAATACGTAGGGTGCGAGCGTTGTCCGGAATTACTGGGCGTAAAGA

GCTCGTAGGTGGTTTGTCGCGTTGTCCGTGAAATTCCCTGGCTTAACTGGGGGCGTGCGGGCGATACGGG

CAGACTGGAGTACTGCAGGGGAGACTGGAATTCCTGGTGTAGCGGTGGAATGCGCAGATATCAGGAGGAA

CACCGGTGGCGAAGGCGGGTCTCTGGGCAGTAACTGACGCTGAGGAGCGAAAGCGTGGGGAGCGAACAGG

ATTAGATACCCTGGTAGTCC

>OTU_56

CAAGCTACGTGCCAGCAGCGGCGGTAATACGTAGGGTGCAAGCGTTAATCGGAATTACTGGGCGTAAAGC

GTGCGCAGGCGGTTTTGTAAGACAGAGGTGAAATCCCCGGGCTCAACCTGGGAACTGCCTTTGTGACTGC

AAGGCTGGAGTGCGGCAGAGGGGGATGGAATTCCGCGTGTAGCAGTGAAATGCGTAGATATGCGGAGGAA

CACCGATGGCGAAGGCAATCCCCTGGGCCTGCACTGACGCTCATGCACGAAAGCGTGGGGAGCAAACAGG

ATTAGATACCCTGGTAGTCC

>OTU_57

CAAGCTTGGTGCCAGCAGCGGCGGTAATACGGAGGGGGCTAGCGTTGTTCGGAATTACTGGGCGTAAAGC

GCACGTAGGCGGCTTTGTAAGTTAGAGGTGAAAGCCTGGAGCTCAACTCCAGAACTGCCTTTAAGACTGC

ATCGCTTGAATCCAGGAGAGGTGAGTGGAATTCCGAGTGTAGAGGTGAAATTCGTAGATATTCGGAAGAA

CACCAGTGGCGAAGGCGGCTCACTGGACTGGTATTGACGCTGAGGTGCGAAAGCGTGGGGAGCAAACAGG

ATTAGATACCCTGGTAGTCC

>OTU_58

CAACCTTCGTGCCAGCAGCGGCGGTAATACGTAGGGCGCAAGCGTTGTCCGGAATTATTGGGCGTAAAGA

GCTCGTAGGCGGTTTGTCGCGTCTGCTGTGAAAGCCCGGGGCTTAACCCCGGGTGTGCAGTGGGTACGGG

CAGACTTGAGTGCAGTAGGGGAGACTGGAATTCCTGGTGTAGCGGTGAAATGCGCAGATATCAGGAGGAA

CACCGATGGCGAAGGCAGGTCTCTGGGCTGTTACTGACGCTGAGGAGCGAAAGCATGGGGAGCGAACAGG

ATTAGATACCCTGGTAGTCC

>OTU_59

CAACGAAGGTGCCAGCAGCGGCGGTAATACGAAGGGGGCTAGCGTTGCTCGGAATCACTGGGCGTAAAGG

GCGCGTAGGCGGCTGATTTAGTCGAGGGTGAAAGCCCGTGGCTCAACCACGGAATGGCCTTCGATACTGA

TTGGCTCGAGACCGGAAGAGGACAGCGGAACTGCGAGTGTAGAGGTGAAATTCGTAGATATTCGCAAGAA

CACCAGTGGCGAAGGCGGCTGTCTGGTCCGGTTCTGACGCTGAGGCGCGAAAGCGTGGGGAGCAAACAGG

ATTAGATACCCTGGTAGTCC

>OTU_60

CAACTCGAGTGCCAGCAGCGGCGGTAATACGTAGGTGGCAAGCGTTGTCCGGATTTATTGGGCGTAAAGC

GAGTGCAGGCGGCTCGATAAGTCTGATGTGAAAGCCTTCGGCTCAACCGGAGAATTGCATCAGAAACTGT

CGAGCTTGAGTACAGAAGAGGAGAGTGGAACTCCATGTGTAGCGGTGAAATGCGTAGATATATGGAAGAA

CACCGGTGGCGAAGGCGGCTCTCTGGTCTGTTACTGACGCTGAGGCTCGAAAGCATGGGTAGCGAACAGG

ATTAGATACCCTGGTAGTCC

>OTU_61

CAACTCGAGTGCCAGCAGCGGCGGTAATACGGAGGATCCGAGCGTTATCCGGATTTATTGGGTTTAAAGG

GAGCGTAGGTGGATTGTTAAGTCAGTTGTGAAAGTTTGCGGCTCAACCGTAAAATTGCAGTTGAAACTGG

CAGTCTTGAGTACAGTAGAGGTGGGCGGAATTCGTGGTGTAGCGGTGAAATGCTTAGATATCACGAAGAA

CTCCGATTGCGAAGGCAGCTCACTAGACTGCAACTGACACTGATGCTCGAAAGTGTGGGTATCAAACAGG

ATTAGATACCCTGGTAGTCC

>OTU_62

CACAACTGGTGCCAGCAGCTGCGGTAATACGTAGGGTGCGAGCGTTATCCGGAATTATTGGGCGTAAAGA

GCTCGTAGGCGGTTTGTCGCGTCTGTCGTGAAAGTCCGGGGCTTAACCCCGGATCTGCGGTGGGTACGGG

CAGACTAGAGTGCAGTAGGGGAGACTGGAATTCCTGGTGTAGCGGTGGAATGCGCAGATATCAGGAGGAA

CACCGATGGCGAAGGCAGGTCTCTGGGCTGTAACTGACGCTGAGGAGCGAAAGCATGGGGAGCGAACAGG

ATTAGATACCCTGGTAGTCC

>OTU_63

CAACACGTGTGCCAGCAGCGGCGGTAATACGTAGGGGGCGAGCGTTGTCCGGAATTATTGGGCGTAAAGC

GCGCGCAGGCGGTCTCTTAAGTCTGATGTGAAAGCCCACGGCTCAACCGTGGAGGGTCATTGGAAACTGG

GGGACTTGAGGGCAGGAGAGGAGAGCGGAATTCCACGTGTAGCGGTGAAATGCGTAGAGATGTGGAGGAA

CACCAGTGGCGAAGGCGGCTCTCTGGCCTGCACCTGACGCTGAGGCGCGAAAGCGTGGGGAGCAAACAGG

ATTAGATACCCTGGTAGTCC

>OTU_64

CAACACGTGTGCCAGCAGCGGCGGTAATACGTAGGGTGCGAGCGTTAATCGGAATTACTGGGCGTAAAGC

GTGCGCAGGCGGTTTGGCAAGTCAGATGTGAAATCCCCGAGCTCAACTTGGGAACTGCGTTTGAAACTGC

CAGACTAGAATATGTCAGAGGGGGGTAGAATTCCACGTGTAGCAGTGAAATGCGTAGAGATGTGGAGGAA

TACCAATGGCGAAGGCAGCCCCCTGGGATAATATTGACGCTCATGCACGAAAGCGTGGGGAGCAAACAGG

ATTAGATACCCTGGTAGTCC

>OTU_65

CAAGCATCGTGCCAGCAGCGGCGGTAATACGTAGGGGGCAAGCGTTATCCGGATTTACTGGGTGTAAAGG

GAGCGTAGGCGGCGATGCAAGTCAGAAGTGAAAGCCCGGGGCTCAACTCCGGGACTGCTTTTGAAACTGT

GTTGCTAGATTGCAGGAGAGGTAAGTGGAATTCCTAGTGTAGCGGTGAAATGCGTAGATATTAGGAGGAA

CACCAGTGGCGAAGGCGGCTTACTGGACTGTAAATGACGCTGAGGCTCGAAAGCGTGGGGAGCAAACAGG

ATTAGATACCCTGGTAGTCC

>OTU_66

CAAGAGGTGTGCCAGCAGCGGCGGTAATACGTAGGGTGCAAGCGTTAATCGGAATTACTGGGCGTAAAGC

GTGCGCAGGCGGTTGTGTAAGACAGGCGTGAAATCCCCGGGCTCAACCTGGGAATGGCGCTTGTGACTGC

ACAACTGGAGTGCGGCAGAGGGGGATGGAATTCCGCGTGTAGCAGTGAAATGCGTAGATATGCGGAGGAA

CACCGATGGCGAAGGCAATCCCCTGGGCCTGCACTGACGCTCATGCACGAAAGCGTGGGGAGCAAACAGG

ATTAGATACCCTGGTAGTCC

>OTU_67

CAACCTTCGTGCCAGCAGCGGCGGTAATACGAAGGGAGCTAGCGTTGTTCGGAATCACTGGGCGTAAAGC

GCACGTAGACGGTTTATCAAGTTGGGAGTGAAATCCCGGGGCTTAACCTCGGAATTGCTTCCAAAACTGG

TTGACTAGAGGTCGGTAGGGGATAGTGGAATTCCTAGTGTAGAGGTGAAATTCTTAGATATTAGGAGGAA

CACCGGTGGCGAAGGCGACTATCTGGACCGATTCTGACGTTGAGGTGCGAAAGCGTGGGGAGCAAACAGG

ATTAGATACCCTGGTAGTCC

>OTU_68

CAAGAGCAGTGCCAGCAGCGGCGGTAATACGTAGGGTGCGAGCGTTAATCGGATTTACTGGGCGTAAAGC

GTGCGCAGGCGGCCCGTTAAGACAGGTGTGAAATCCCTGGGCTCAACCTAGGAATTGCGCTTGTGACTGG

CGGGCTCGAGTACGGTAGAGGGGGGTGGAATTCCTGGTGTAGCAGTGAAATGCGTAGAGATCAGGAGGAA

CACCGATGGCGAAGGCAGCCCCCTGGGCCTGTACTGACGCTCATGCACGAAAGCGTGGGGAGCAAACAGG

ATTAGATACCCTGGTAGTCC

>OTU_69

CAACTCGAGTGCCAGCAGCAGCGGTAATACGGAGGGGGTTAGCGTTGTTCGGAATGACTGGGCGTAAAGC

GCGCGTAGGCGGATCGGTAAGTTGGGGGTGAAAGCCCAGGGCTCAACCCTGGAACGGCCTTCAAGACTCC

CGGTCTGGAGTTCGAGAGAGGTGGGTGGAATTCCGAGTGTAGAGGTGAAATTCGTAGATATTCGGAGGAA

CACCAGTGGCGAAGGCGGCCCACTGGCTCGACACTGACGCTGAGGCGCGAAAGCGTGGGGAGCAAACAGG

ATTAGATACCCTGGTAGTCC

>OTU_70

CAACTCCTGTGCCAGCAGCGGCGGTAATACGTATGGTGCAAGCGTTATCCGGATTTACTGGGTGTAAAGG

GAGCGTAGACGGAGTGGCAAGTCTGATGTGAAAACCCGGGGCTCAACCCCGGGACTGCATTGGAAACTGT

CAATCTAGAGTACCGGAGAGGTAAGCGGAATTCCTAGTGTAGCGGTGAAATGCGTAGATATTAGGAGGAA

CACCAGTGGCGAAGGCGGCTTACTGGACGGTAACTGACGTTGAGGCTCGAAAGCGTGGGGAGCAAACAGG

ATTAGATACCCTGGTAGTCC

>OTU_71

CAACTGGTGTGCCAGCAGCGGCGGTAATACGTAGGTGGCGAGCGTTATCCGGATTTACTGGGCGTAAAGG

GAGCGTAGGCGGATGATTAAGTGGGATGTGAAATACCCGGGCTCAACTTGGGTGCTGCATTCCAAACTGG

TTATCTAGAGTGCAGGAGAGGAGAGTGGAATTCCTAGTGTAGCGGTGAAATGCGTAGAGATTAGGAAGAA

CACCAGTGGCGAAGGCGACTCTCTGGACTGTAACTGACGCTGAGGCTCGAAAGCGTGGGGAGCAAACAGG

ATTAGATACCCTGGTAGTCC

>OTU_72

CAACTCGAGTGCCAGCAGCGGCGGTAATACGTAGGGCGCAAGCGTTGTCCGGAATTATTGGGCGTAAAGA

GCTCGTAGGCGGTTTGTCGCGTCTGCCGTGAAAACCTAGTGCTTAACACTGGGCGTGCGGTGGGTACGGG

CAGGCTAGAGTGCGGTAGGGGAGACTGGAATTCCTGGTGTAGCGGTGGAATGCGCAGATATCAGGAGGAA

CACCGATGGCGAAGGCAGGTCTCTGGGCCGTTACTGACGCTGAGGAGCGAAAGCATGGGTAGCGAACAGG

ATTAGATACCCTGGTAGTCC

>OTU_73

CACAACTGGTGCCAGCAGCTGCGGTAATACGTAGGGTGCAAGCGTTGTCCGGAATTACTGGGCGTAAAGA

GCTCGTAGGTGGTTTGTCACGTCGTCTGTGAAATTCCACAGCTTAACTGTGGGCGTGCAGGCGATACGGG

CTGACTTGAGTACTGTAGGGGTAACTGGAATTCCTGGTGTAGCGGTGAAATGCGCAGATATCAGGAGGAA

CACCGATGGCGAAGGCAGGTTACTGGGCAGTTACTGACGCTGAGGAGCGAAAGCATGGGTAGCAAACAGG

ATTAGATACCCTGGTAGTCC

>OTU_74

CAACCTTCGTGCCAGCAGCTGCGGTAATACAGAGGGTGCAAGCGTTAATCGGAATTACTGGGCGTAAAGC

GCGCGTAGGTGGTTAGTTAAGTTGGATGTGAAATCCCCGGGCTCAACCTGGGAACTGCATTCAAAACTGA

CTGACTAGAGTATGGTAGAGGGTGGTGGAATTTCCTGTGTAGCGGTGAAATGCGTAGATATAGGAAGGAA

CACCAGTGGCGAAGGCGACCACCTGGACTGATACTGACACTGAGGTGCGAAAGCGTGGGGAGCAAACAGG

ATTAGATACCCTGGTAGTCC

>OTU_75

CAACGATCGTGCCAGCAGCGGCGGTAATACGGAGGGTGCGAGCGTTAATCGGAATTACTGGGCGTAAAGC

GCGCGTAGGCGGCGTGATAAGCCGGTTGTGAAAGCCCCGGGCTCAACCTGGGAACGGCATCCGGAACTGT

CAGGCTAGAGTGCAGGAGAGGAAGGTAGAATTCCCGGTGTAGCGGTGAAATGCGTAGAGATCGGGAGGAA

TACCAGTGGCGAAGGCGGCCTTCTGGACTGACACTGACGCTGAGGTGCGAAAGCGTGGGTAGCAAACAGG

ATTAGATACCCTGGTAGTCC

>OTU_76

CACAGACTGTGCCAGCAGCCGCGGTAATACGAAGGGGGCTAGCGTTGTTCGGATTTACTGGGCGTAAAGC

GCACGTAGGCGGGCTTTTAAGTCAGGGGTGAAATCCCGGGGCTCAACCCCGGAACTGCCTTTGATACTGG

AAGTCTTGAGTATGGAAGAGGTGAGTGGAATTCCGAGTGTAGAGGTGAAATTCGTAGATATTCGGAGGAA

CACCAGTGGCGAAGGCGGCTCACTGGTCCATTACTGACGCTGAGGTGCGAAAGCGTGGGGAGCAAACAGG

ATTAGATACCCTGGTAGTCC

>OTU_77

CACAGACTGTGCCAGCAGCAGCGGTAATACGTAGGTGGCAAGCGTTGTCCGGATTTATTGGGCGTAAAGC

GCGCGCAGGCGGTCCTTTAAGTCTGATGTGAAATCTTGCGGCTCAACCGCAAGCGGTCATTGGAAACTGG

GGGACTTGAGTGCAGAAGAGGAAAGCGGAATTCCACGTGTAGCGGTGAAATGCGTAGAGATGTGGAGGAA

CACCAGTGGCGAAGGCGGCTTTCTGGTCTGTAACTGACGCTGAGGCGCGAAAGCGTGGGGAGCAAACAGG

ATTAGATACCCTGGTAGTCC

>OTU_78

CAAGAGGTGTGCCAGCAGCTGCGGTAATACGGAGGGGGCTAGCGTTGTTCGGAATTACTGGGCGTAAAGC

GCACGTAGGCGGCTTTGTAAGTCAGGGGTGAAAGCCTGGAGCTCAACTCCAGAACTGCCTTTGAGACTGC

ATCGCTTGAATCCGGGAGAGGTGAGTGGAATTCCGAGTGTAGAGGTGAAATTCGTAGATATTCGGAAGAA

CACCAGTGGCGAAGGCGGCTCACTGGACCGGTATTGACGCTGAGGTGCGAAAGCGTGGGGAGCAAACAGG

ATTAGATACCCTGGTAGTCC

>OTU_79

CAAGAGCAGTGCCAGCAGCGGCGGTAATACGTAGGGTGCAAGCGTTGTCCGGAATTATTGGGCGTAAAGA

GCTCGTAGGCGGCTTGTCGCGTCTGCTGTGAAAATCCGGGGCTTAACTCCGGACCTGCAGTGGATACGGG

CAGGCTTGAGTTCGGTAGGGGAGACTGGAATTCCTGGTGTAGCGGTGAAATGCGCAGATATCAGGAGGAA

CACCGGTGGCGAAGGCGGGTCTCTGGGCCGATACTGACGCTGAGGAGCGAAAGCGTGGGGAGCGAACAGG

ATTAGATACCCTGGTAGTCC

>OTU_80

CAACTCGAGTGCCAGCAGCGGCGGTAATACGGAGGATCCGAGCGTTATCCGGATTTATTGGGTTTAAAGG

GAGCGTAGGCGGGTTGTTAAGTCAGTTGTGAAAGTTTGCGGCTCAACCGTAAAATTGCAGTTGATACTGG

CGACCTTGAGTGCAACAGAGGTAGGCGGAATTCGTGGTGTAGCGGTGAAATGCTTAGATATCACGAAGAA

CTCCGATTGCGAAGGCAGCTTACTGGATTGTAACTGACGCTGATGCTCGAAAGTGTGGGTATCAAACAGG

ATTAGATACCCTGGTAGTCC

>OTU_81

CAACACGTGTGCCAGCAGCAGCGGTAATACAGAGGGTGCAAGCGTTAATCGGATTTACTGGGCGTAAAGC

GCGCGTAGGTGGCCAATTAAGTCAAATGTGAAATCCCCGAGCTTAACTTGGGAATTGCATTCGATACTGG

TTGGCTAGAGTATGGGAGAGGATGGTAGAATTCCAGGTGTAGCGGTGAAATGCGTAGAGATCTGGAGGAA

TACCGATGGCGAAGGCAGCCATCTGGCCTAATACTGACACTGAGGTGCGAAAGCATGGGGAGCAAACAGG

ATTAGATACCCTGGTAGTCC

>OTU_82

CACAACTGGTGCCAGCAGCCGCGGTAATACGAAGGGGGCTAGCGTTGCTCGGAATTACTGGGCGTAAAGG

GCGCGTAGGCGGATCGTTAAGTCAGAGGTGAAATCCCAGGGCTCAACCCTGGAACTGCCTTTGATACTGG

CGATCTTGAGTATGAGAGAGGTATGTGGAACTCCGAGTGTAGAGGTGAAATTCGTAGATATTCGGAAGAA

CACCAGTGGCGAAGGCGACATACTGGCTCATTACTGACGCTGAGGCGCGAAAGCGTGGGGAGCAAACAGG

ATTAGATACCCTGGTAGTCC

>OTU_83

CAAGCATCGTGCCAGCAGCGGCGGTAATACGTAGGTGGCAAGCGTTATCCGGATTTATTGGGCGTAAAGC

GAGCGCAGGCGGTTGCTTAGGTCTGATGTGAAAGCCTTCGGCTTAACCGAAGAAGTGCATCGGAAACCGG

GCGACTTGAGTGCAGAAGAGGACAGTGGAACTCCATGTGTAGCGGTGGAATGCGTAGATATATGGAAGAA

CACCAGTGGCGAAGGCGGCTGTCTGGTCTGCAACTGACGCTGAGGCTCGAAAGCATGGGTAGCGAACAGG

ATTAGATACCCTGGTAGTCC

>OTU_84

CAAGCAAGGTGCCAGCAGCTGCGGTAATACAGAGGGTGCAAGCGTTAATCGGAATTACTGGGCGTAAAGC

GAGCGTAGGTGGCTTAATAAGTCAGATGTGAAATCCCCGGGCTTAACCTGGGAACTGCATCTGATACTGT

TAGGCTAGAGTAGGTGAGAGGGAGGTAGAATTTCAGGTGTAGCGGTGAAATGCGTAGAGATCTGAAGGAA

TACCGATGGCGAAGGCAGCCTCCTGGCATCATACTGACACTGAGGTTCGAAAGCGTGGGTAGCAAACAGG

ATTAGATACCCTGGTAGTCC

>OTU_85

CACAGACTGTGCCAGCAGCGGCGGTAATACGTAGGGCGCAAGCGTTGTCCGGAATTATTGGGCGTAAAGA

GCTCGTAGGCGGTCTGTCGCGTCTGCTGTGAAATCCCGAGGCTCAACCTCGGGCTTGCAGTGGGTACGGG

CAGACTAGAGTGCGGTAGGGGAGAATGGAATTCCTGGTGTAGCGGTGGAATGCGCAGATATCAGGAGGAA

CACCGATGGCGAAGGCAGTTCTCTGGGCCGTAACTGACGCTGAGGAGCGAAAGCGTGGGGAGCGAACAGG

ATTAGATACCCTGGTAGTCC

>OTU_86

CAACTGCAGTGCCAGCAGCTGCGGTAATACAGAGGGTGCAAGCGTTGTTCGGAATTACTGGGCGTAAAGC

GTGCGTAGTCGGTATTGAGAGTCACGGGTGAAATCCCAGGGCTTAACCCTGGAACTGCCTGTGAGACCTC

AGTACTAGAGTGTGAGAGGGGATAGTGGAATACCCAGTGTAGCGGTGAAATGCGTAGAGATTGGGTGGAA

CACCGGTGGCGAAGGCGGCTATCTGGCTCACAACTGACGATCAGGCACGAAAGCGTGGGGAACAAACAGG

ATTAGATACCCTGGTAGTCC

>OTU_87

CAAGACGAGTGCCAGCAGCGGCGGTGATACGTAGGGTCCGAGCGTTGTCCGGAATTATTGGGCGTAAAGG

GCTTGTAGGCGGTCTGTCGCGTCGGAAGTGAAAACTCAGGGCTTAACCCTGAGCCTGCTTCCGATACGGG

CAGACTAGAGGAATGCAGGGGAGAATGGAATTCCTGGTGGAGCGGTGGAATGCGCAGATATCAGGAGGAA

CACCAGTGGCGAAGGCGGTTCTCTGGGCATTTCCTGACGCTGAGAAGCGAAAGCGTGGGGAGCAAACAGG

CTTAGATACCCTGGTAGTCC

>OTU_88

CAAGAGCAGTGCCAGCAGCGGCGGTAATACGTAGGTGGCAAGCGTTGTCCGGAATTATTGGGCGTAAAGC

GCGCGCAGGCGGTCCTTTAAGTCTGATGTGAAAGCCCACGGCTCAACCGTGGAGGGTCATTGGAAACTGG

GGGACTTGAGTGCAGAAGAGGAGAGCGGAATTCCACGTGTAGCGGTGAAATGCGTAGAGATGTGGAGGAA

CACCAGTGGCGAAGGCGGCTCTCTGGTCTGTAACTGACGCTGAGGCGCGAAAGCGTGGGGAGCAAACAGG

ATTAGATACCCTGGTAGTCC

>OTU_89

CAACTCGAGTGCCAGCAGCGGCGGTAATACGTAGGTGGCAAGCGTTGTCCGGATTTATTGGGCGTAAAGC

GAGCGCAGGCGGTCTTTTAAGTCTGATGTGAAAGCCCCCGGCTTAACCGGGGAGGGTCATTGGAAACTGG

GAGACTTGAGTGCAGAAGAGGAAAGCGGAATTCCATGTGTAGCGGTGAAATGCGTAGATATATGGAGGAA

CACCAGTGGCGAAGGCGGCTTTCTGGTCTGTAACTGACGCTGAGGCTCGAAAGCGTGGGGAGCAAACAGG

ATTAGATACCCTGGTAGTCC

>OTU_90

CAAGCAAGGTGCCAGCAGCGGCGGTAATACAGAGGGTGCAAGCGTTAATCGGAATTACTGGGCGTAAAGC

GCGCGTAGGTGGTTATTTAAGTCAGATGTGAAAGCCCCGGGCTTAACCTGGGAACTGCATCTGATACTGG

ATAACTAGAGTAGGTGAGAGGGGAGTAGAATTCCAGGTGTAGCGGTGAAATGCGTAGAGATCTGGAGGAA

TACCGATGGCGAAGGCAGCTCCCTGGCATCATACTGACACTGAGGTGCGAAAGCGTGGGTAGCAAACAGG

ATTAGATACCCTGGTAGTCC

>OTU_91

CAAGCTTGGTGCCAGCAGCGGCGGTAATACGTAGGTGGCAAGCGTTGTCCGGATTTATTGGGCGTAAAGC

GAGCGCAGGCGGTTTCTTAAGTCTGATGTGAAAGCCTTCGGCTCAACCGAAGAAGTGCATCGGAAACTGG

GAAACTTGAGTGCAGAAGAGGACAGTGGAACTCCATGTGTAGCGGTGAAATGCGTAGATATATGGAAGAA

CACCAGTGGCGAAGGCGGCTGTCTGGTCTGTAACTGACGCTGAGGCTCGAAAGCATGGGTAGCAAACAGG

ATTAGATACCCTGGTAGTCC

>OTU_92

CAACCTAGGTGCCAGCAGCGGCGGTAATACGAAGGGGGCTAGCGTTGCTCGGAATCACTGGGCGTAAAGG

GCGCGTAGGCGGACTCTTAAGTCGGAGGTGAAAGCCCAGGGCTCAACCCTGGAATTGCCTTCGATACTGG

GAGTCTTGAGTTCGGAAGAGGTTGGTGGAACTGCGAGTGTAGAGGTGAAATTCGTAGATATTCGCAAGAA

CACCAGTGGCGAAGGCGGCCAACTGGTCCGATACTGACGCTGAGGCGCGAAAGCGTGGGGAGCAAACAGG

ATTAGATACCCTGGTAGTCC

>OTU_93

CAAGAGCAGTGCCAGCAGCAGCGGTAATACGTAGGGTGCGAGCGTTGTCCGGAATTATTGGGCGTAAAGA

GCTTGTAGGCGGTCTGTCGCGTCTGCTGTGAAAGACCGGGGCTTAACTCCGGTTCTGCAGTGGGTACGGG

CAGACTAGAGTGTGGTAGGGGAGACTGGAATTCCTGGTGTAGCGGTGAAATGCGCAGATATCAGGAGGAA

CACCGATGGCGAAGGCAGGTCTCTGGGCCATTACTGACGCTGAGAAGCGAAAGCATGGGGAGCGAACAGG

ATTAGATACCCTGGTAGTCC

>OTU_94

CAAGTCGTGTGCCAGCAGCGGCGGTAATACGTAGGTGGCAAGCGTTGTCCGGAATTATTGGGCGTAAAGC

GCGCGCAGGTGGTTTAATAAGTCTGATGTGAAAGCCCACGGCTCAACCGTGGAGGGTCATTGGAAACTGT

TAAACTTGAGTGCAGGAGAGAAAAGTGGAATTCCTAGTGTAGCGGTGAAATGCGTAGAGATTAGGAGGAA

CACCAGTGGCGAAGGCGGCTTTTTGGCCTGTAACTGACACTGAGGCGCGAAAGCGTGGGGAGCAAACAGG

ATTAGATACCCTGGTAGTCC

>OTU_95

CAACGATCGTGCCAGCAGCAGCGGTAATACGAAGGGGGCTAGCGTTGCTCGGAATCACTGGGCGTAAAGG

GCGCGTAGGCGGCCATTCAAGTCGGGGGTGAAAGCCTGTGGCTCAACCACAGAATTGCCTTCGATACTGT

TTGGCTTGAGTTTGGTAGAGGTTGGTGGAACTGCGAGTGTAGAGGTGAAATTCGTAGATATTCGCAAGAA

CACCAGTGGCGAAGGCGGCCAACTGGACCAATACTGACGCTGAGGCGCGAAAGCGTGGGGAGCAAACAGG

ATTAGATACCCTGGTAGTCC

>OTU_96

CAAGAGCAGTGCCAGCAGCGGCGGTAATTCCAGCTCCAATAGCGTATATTTAAGTTGTTGCAGTTAAAAA

GCTCGTAGTTGGATCTTGGGTCGTAAAGGTCGGTCCGCCTACTCGGTGTGCACCTGCCCTTCCGTCCCTT

TTGTCGGCGGCGTGCTCCTGGCCTTAATTGGCTGGGTCGCGGCTCCGGCGCTGTTACTTTGAAAAAATTA

GAGTGCTCAAAGCAAGCTTATGCTCTGAATACATTAGCATGGAATAACGTGATAGGAGTCTGGTCCTATT

GTGTTGGCCTTCGGGACCGG

>OTU_97

CACAGACTGTGCCAGCAGCCGCGGTAATACGGAGGGTGCAAGCGTTACCCGGAATCACTGGGCGTAAAGG

GCGTGTAGGCGGAATGTTAAGTCTGGTTTTAAAGACTGGGGCTCAACCCCAGGAGTGGACTGGATACTGG

CAATCTTGACCTCTGGAGAGGTAACTGGAATTCCTGGTGTAGCGGTGGAATGCGTAGATACCAGGAGGAA

CACCAATGGCGAAGGCAAGTTACTGGACAGAAGGTGACGCTGAGGCGCGAAAGTGTGGGGAGCAAACCGG

ATTAGATACCCGGGTAGTCC

>OTU_98

CACAGACTGTGCCAGCAGCAGCGGTAATACGGAGGGTGCAAGCGTTATCCGGATTTATTGGGTTTAAAGG

GTCCGTAGGCGGATCGGTAAGTCAGTGGTGAAATCTCATAGCTTAACTATGAAACTGCCATTGATACTGT

CGGTCTTGAGTGTATTTGAAGTAGCTGGAATGAGTAGTGTAGCGGTGAAATGCATAGATATTACTCAGAA

CACCAATTGCGAAGGCAGGTTACTAAGATACAACTGACGCTGATGGACGAAAGCGTGGGGAGCGAACAGG

ATTAGATACCCTGGTAGTCC

>OTU_99

CACAGACTGTGCCAGCAGCTGCGGTAATACGTATGTCCCGAGCGTTATCCGGATTTATTGGGCGTAAAGC

GAGCGCAGACGGTTGATTAAGTCTGATGTGAAAGCCCGGAGCTCAACTCCGGAAAGGCATTGGAAACTGG

TCAACTTGAGTGCAGTAGAGGTAAGTGGAACTCCATGTGTAGCGGTGGAATGCGTAGATATATGGAAGAA

CACCAGCGGCGAAGGCGGCTTACTGGACTGTAACTGACGTTGAGGCTCGAAAGTGTGGGTAGCAAACAGG

ATTAGATACCCTGGTAGTCC

>OTU_100

CAACACCAGTGCCAGCCGCGGCGGTAATACGGAGGGTGCAAGCGTTAATCGGAATTACTGGGCGTAAAGC

GCACGCAGGCGGTCTGTTAAGTCAGATGTGAAATCCCCGGGCTCAACCTGGGAACTGCATCTGATACTGG

CAGGCTTGAGTCTCGTAGAGGGGGGTAGAATTCCAGGTGTAGCGGTGAAATGCGTAGAGATCTGGAGGAA

TACCGGTGGCGAAGGCGGCCCCCTGGACGAAGACTGACGCTCAGGTGCGAAAGCGTGGGGAGCAAACAGG

ATTAGATACCCTGGTAGTCC

>OTU_101

CAACGTTGGTGCCAGCAGCTGCGGTAATACGGAGGATCCGAGCGTTATCCGGATTTATTGGGTTTAAAGG

GAGCGTAGGTGGACTGGTAAGTCAGTTGTGAAAGTTTGCGGCTCAACCGTAAAATTGCAGTTGATACTGT

CAGTCTTGAGTACAGTAGAGGTGGGCGGAATTCGTGGTGTAGCGGTGAAATGCTTAGATATCACGAAGAA

CTCCGATTGCGAAGGCAGCTCACTGGACTGCAACTGACACTGATGCTCGAAAGTGTGGGTATCAAACAGG

ATTAGATACCCTGGTAGTCC

>OTU_102

CAAGCATCGTGCCAGCAGCGGCGGTAATACGTAGGGTCCGAGCGTTGTCCGGAATTATTGGGCGTAAAGG

GCTCGTAGGCGGTTTGTCGCGTCGGGAGTGAAAACACCGGGCTTAACTCGGTGCTTGCTTCCGATACGGG

CAGACTGGAGGTATGCAGGGGAGAACGGAATTCCTGGTGTAGCGGTGAAATGCGCAGATATCAGGAGGAA

CACCGGTGGCGAAGGCGGTTCTCTGGGCATTACCTGACGCTGAGGAGCGAAAGTGTGGGGAGCGAACAGG

ATTAGATACCCTGGTAGTCC

>OTU_103

CAACCATGGTGCCAGCAGCGGCGGTAATACGTAGGGTGCGAGCGTTAATCGGAATTACTGGGCGTAAAGC

GAGCGCAGACGGTTACTTAAGCAGGATGTGAAATCCCCGGGCTCAACCTGGGAACTGCGTTCTGAACTGG

GTGACTAGAGTGTGTCAGAGGGAGGTAGAATTCCACGTGTAGCAGTGAAATGCGTAGAGATGTGGAGGAA

TACCGATGGCGAAGGCAGCCTCCTGGGATAACACTGACGTTCATGCTCGAAAGCGTGGGTAGCAAACAGG

ATTAGATACCCTGGTAGTCC

>OTU_104

CAAGGTTCGTGCCAGCAGCAGCGGTAATACGGAGGGAGCTAGCGTTGTTCGGAATTACTGGGCGTAAAGC

GCACGTAGGCGGTTATTTAAGTCAGAGGTGAAAGCCCAGTGCTCAACACTGGAACTGCCTTTGAGACTGG

ATAACTTGAATCCAGGAGAGGTGAGTGGAATTCCGAGTGTAGAGGTGAAATTCGTAGATATTCGGAAGAA

CACCAGTGGCGAAGGCGGCTCACTGGACTGGTATTGACGCTGAGGTGCGAAAGCGTGGGGAGCAAACAGG

ATTAGATACCCTGGTAGTCC

>OTU_105

CAAGAGGTGTGCCAGCAGCAGCGGTAATACGTAGGGTGCGAGCGTTGTCCGGAATTATTGGGCGTAAAGG

GCTCGTAGGCGGTTTGTCGCGTCGGGAGTGAAAACCAGGTGCTTAACACCTGGCTTGCTTTCGATACGGG

CAGACTAGAGGTATTCAGGGGAGAACGGAATTCCTGGTGTAGCGGTGAAATGCGCAGATATCAGGAGGAA

CACCGGTGGCGAAGGCGGTTCTCTGGGAATGACCTGACGCTGAGGAGCGAAAGTGTGGGGAGCGAACAGG

ATTAGATACCCTGGTAGTCC

>OTU_106

CAACTGCAGTGCCAGCAGCGGCGGTAATTCCAGCTCCAATAGCGTATATTAAAGTTGCTGCAGTTAAAAA

GCTCGTAGTTGGATCTTGGGAGCGGACGGGCGGTGGCTCGCCTCGCGGCGGACCGCCCGCCCGCTCCCAA

GATCCAACTACGAGCTTTTTAACTGCAGCAACTTTAATATACGCTATTGGAGCTGGAATTACCGCTGCGG

CTGGCACACGTGTTGTGATCGGAAGACACACGTCTGAACTCCAGTCAC
