# Supplementary material for: Metagenomic analysis unveils the microbial landscape of pancreatic tumors
Source: Front Microbiol. 2023 Dec 21;14:1275374. doi: 10.3389/fmicb.2023.1275374 (PMC10764597; doi:10.3389/fmicb.2023.1275374)
Supplement: Supplementary file 5 [file Table_5.DOCX]

**Table S2:** The qRT-PCR expression of PDL1 gene in tumor tissues normalized by GAPDH and expressed as 2^-ddct^ method.

| **Patients** | **PDL1 fold change** |
| --- | --- |
| T1 | 6.02795 |
| T10 | 0.0446 |
| T12 | 100.427 |
| T14 | 0.3956 |
| T16 | 1.77358 |
| T18 | 2.54871 |
| T2 | 8.33973 |
| T20 | 0.32609 |
| T26 | 0.37199 |
| T3 | 2.32947 |
| T30 | 0.21915 |
| T34 | 0.52365 |
| T36 | 0.45586 |
| T38 | 8.11168 |
| T8 | 2.49666 |
| T22 | 1.86318 |
| T24 | 6.87487 |
| T28 | 56.2325 |
| T32 | 64.7436 |
| T40 | 94.5715 |
